# Supplementary material for: Inhibition of KRAS, MEK and PI3K Demonstrate Synergistic Anti-Tumor Effects in Pancreatic Ductal Adenocarcinoma Cell Lines
Source: Cancers (Basel). 2022 Sep 14;14(18):4467. doi: 10.3390/cancers14184467 (PMC9497071; doi:10.3390/cancers14184467)
Supplement: Supplementary file 1 [file cancers-14-04467-s001.zip › cancers-1910357-supplementary.pdf]

Supplementary Figure S1

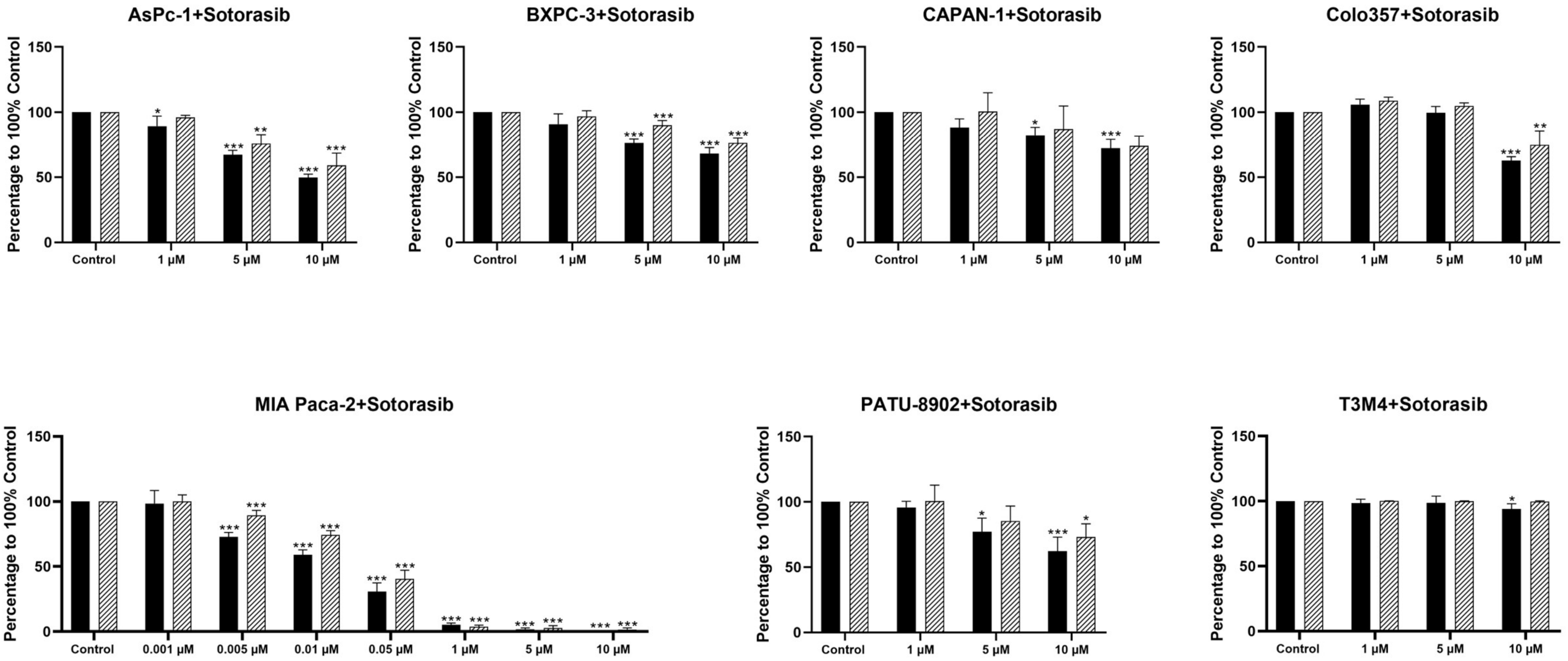

Supplementary Figure S1. Cell viability after 72 hours Sotorasib exposure in PDAC cell lines. Data are presented as Mean ± SD. Significance of a treatment effect (which showed in bar charts) compared to the DMSO control was determined by one-way ANOVA and displayed as *: *P*< 0.033, **: *P*< 0.002, ***: *P*<0.001 (n ≥ 3).

Supplementary Figure S2

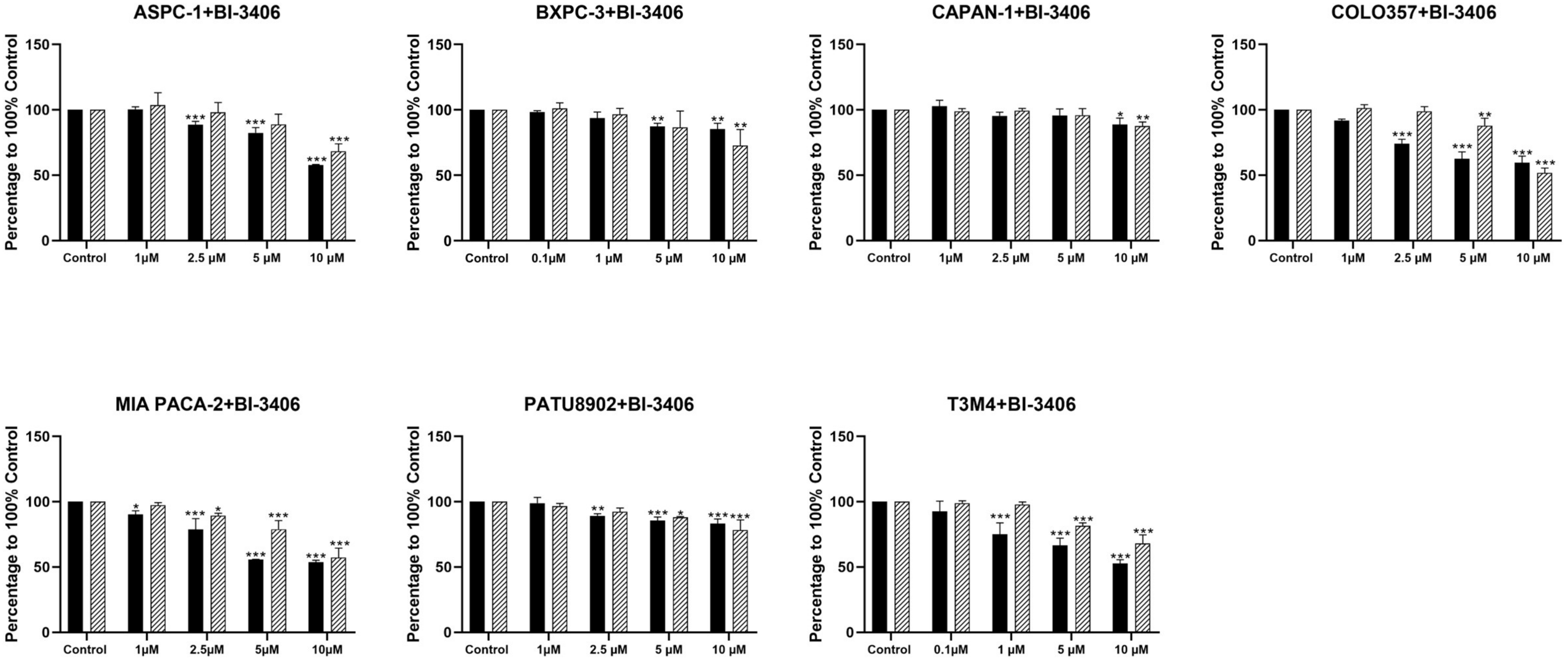

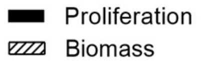

Supplementary Figure S2. Cell viability after 72 hours BI-3406 exposure in PDAC cell lines. Data are presented as Mean ± SD. Significance of a treatment effect (which showed in bar charts) compared to the DMSO control was determined by one-way ANOVA and displayed as *: $P< 0.033$, **: $P< 0.002$, ***: $P<0.001$ ($n \geq 3$).

Supplementary Figure S3

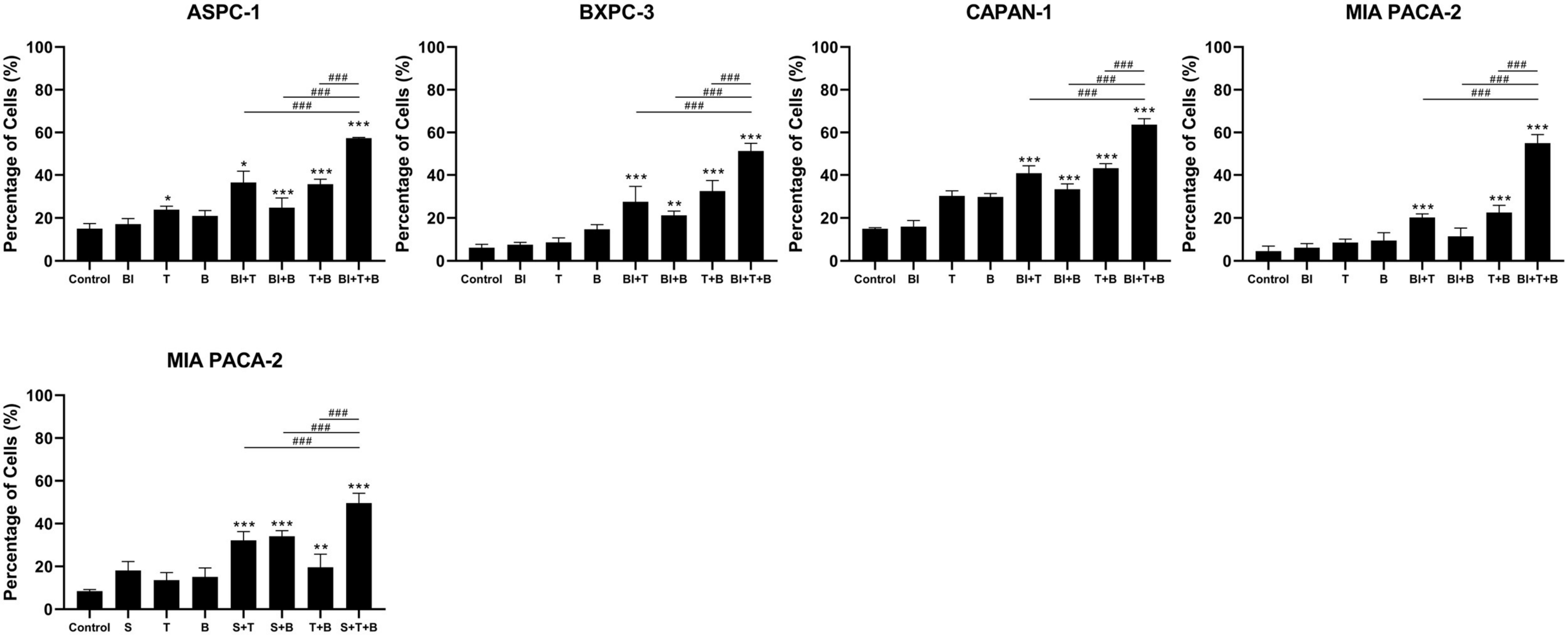

Supplementary Figure S3. PDAC Cell death induction after 72 hours BI-3406, Sotorasib, Trametinib, Buparlisib or inhibitors combination exposure. Data are presented as Mean ± SD. Significance of a treatment effect compared to the DMSO control was determined by one-way ANOVA and displayed as *: *P*< 0.033, **: *P*< 0.002, ***: *P*<0.001 (n ≥ 3). The significance of the treatment effect for double inhibition compared to triple inhibition was determined by one-way ANOVA and is shown as #: P<0.033; ##: P<0.002, ###: P<0.001.
BI: BI-3406; S: Sotorasib; T: Trametinib; B: Buparlisib.

Supplementary Figure S4-1

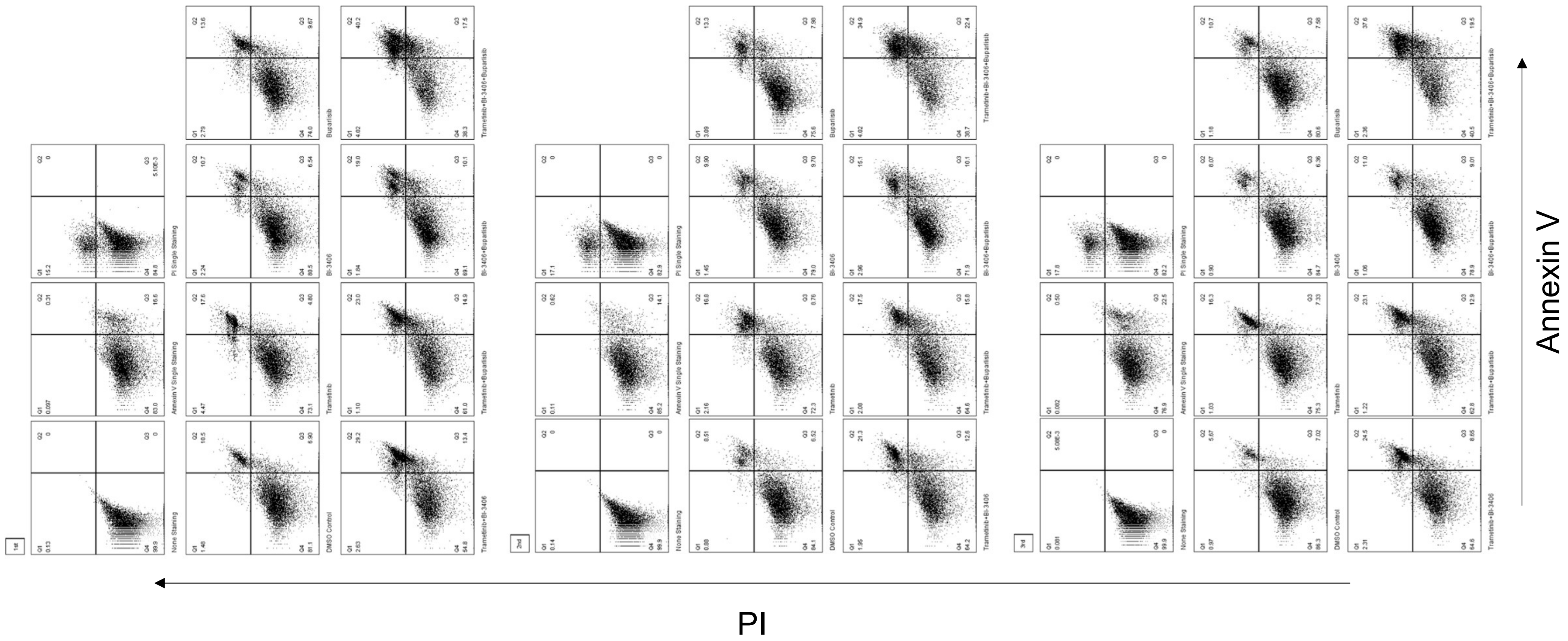

Supplementary Figure S4-1. Apoptosis/necrosis dot plot of ASPC-1 after 72 hours BI-3406, Trametinib, Buparlisib and inhibitor combination exposure.
Q4: Annexin V-/PI-, viable cells
Q3: Annexin V+/PI-, early apoptotic cells
Q2: Annexin V+/PI+, late apoptotic/necrotic cells
Cell death = Q2+Q3

Supplementary Figure S4-2

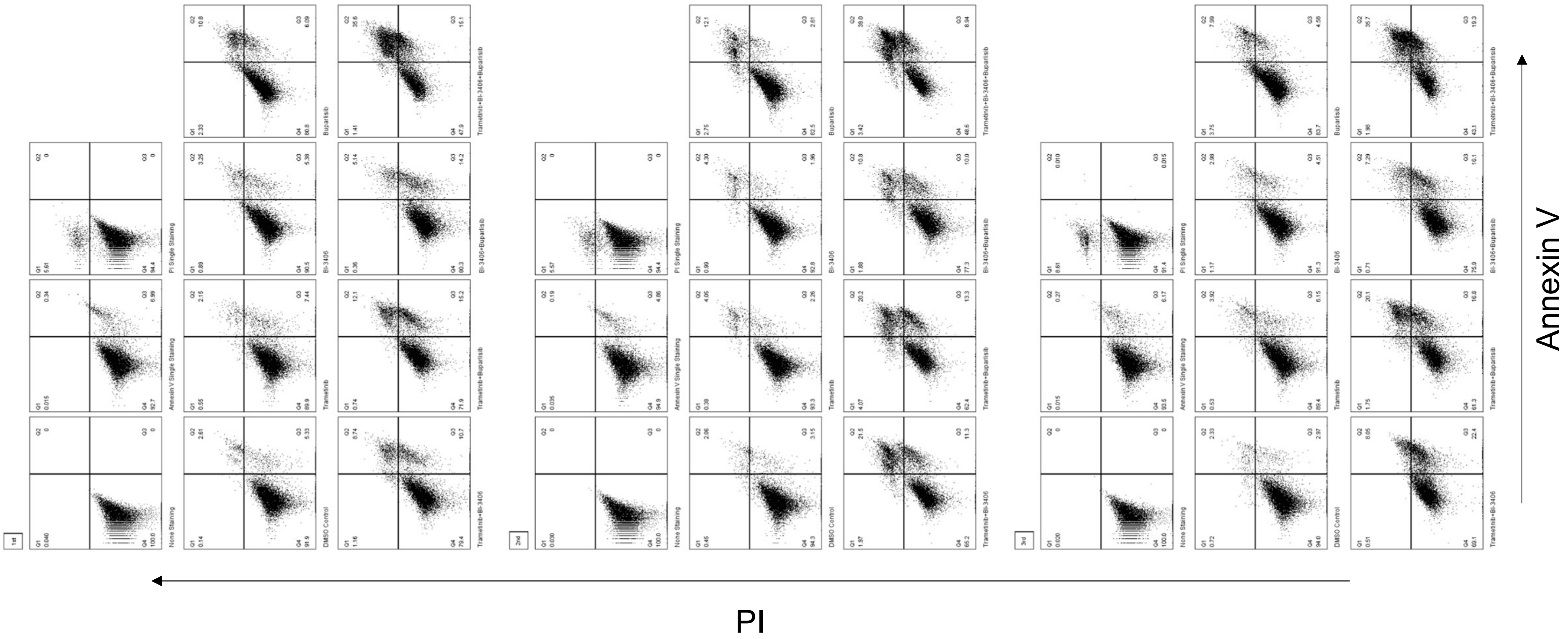

Supplementary Figure S4-2. Apoptosis/necrosis dot plot of BXPC-3 after 72 hours BI-3406, Trametinib, Buparlisib and inhibitor combination exposure.
Q4: Annexin V-/PI-, viable cells
Q3: Annexin V+/PI-, early apoptotic cells
Q2: Annexin V+/PI+, late apoptotic/necrotic cells
Cell death = Q2+Q3

Supplementary Figure S4-3

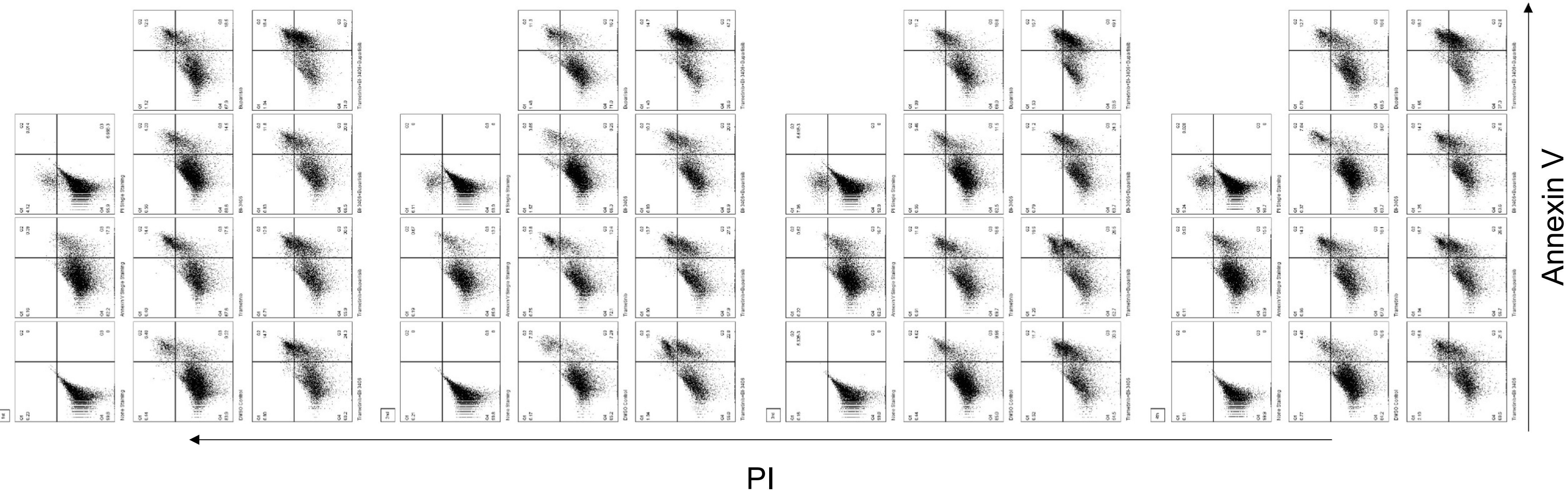

Supplementary Figure S4-3. Apoptosis/necrosis dot plot of CAPAN-1 after 72 hours BI-3406, Trametinib, Buparlisib and inhibitor combination exposure.
Q4: Annexin V-/PI-, viable cells
Q3: Annexin V+/PI-, early apoptotic cells
Q2: Annexin V+/PI+, late apoptotic/necrotic cells
Cell death = Q2+Q3

Supplementary Figure S4-4

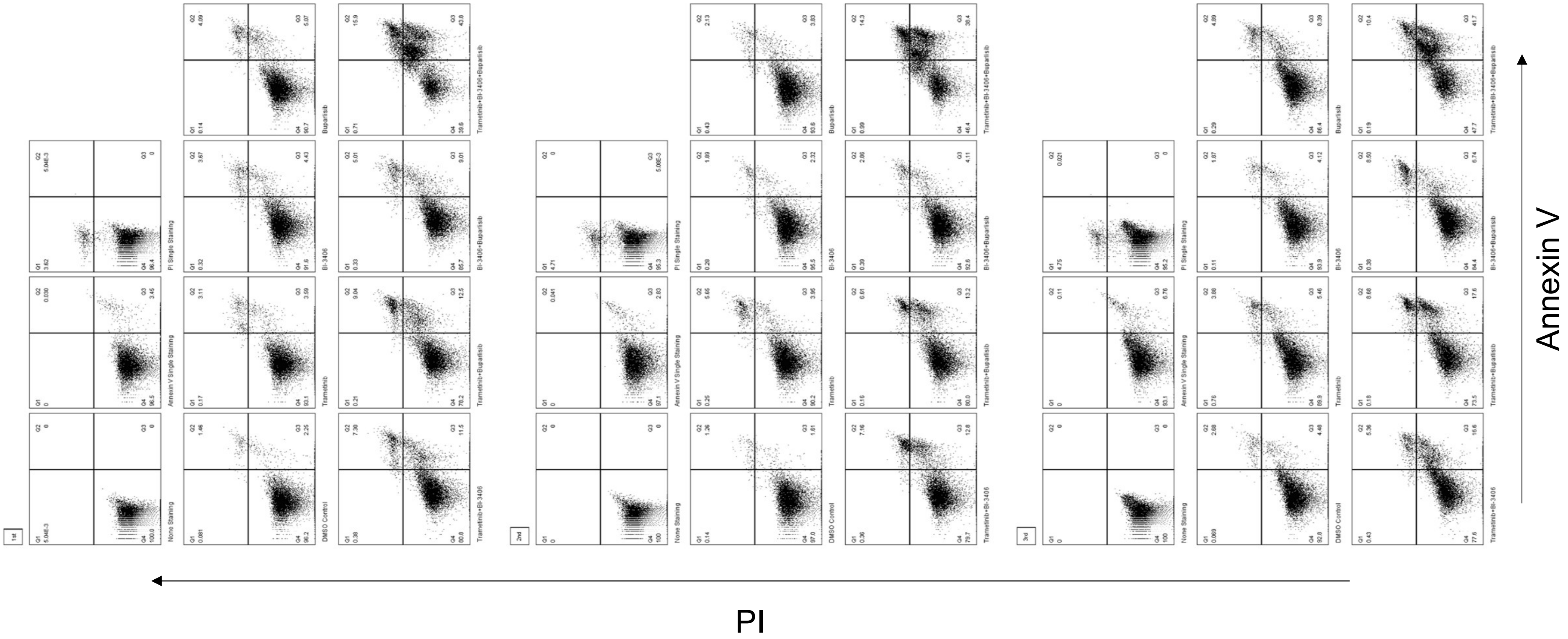

Supplementary Figure S4-4. Apoptosis/necrosis dot plot of MIA PACA-2 after 72 hours BI-3406, Trametinib, Buparlisib and inhibitor combination exposure.
Q4: Annexin V-/PI-, viable cells
Q3: Annexin V+/PI-, early apoptotic cells
Q2: Annexin V+/PI+, late apoptotic/necrotic cells
Cell death = Q2+Q3

Supplementary Figure S4-5

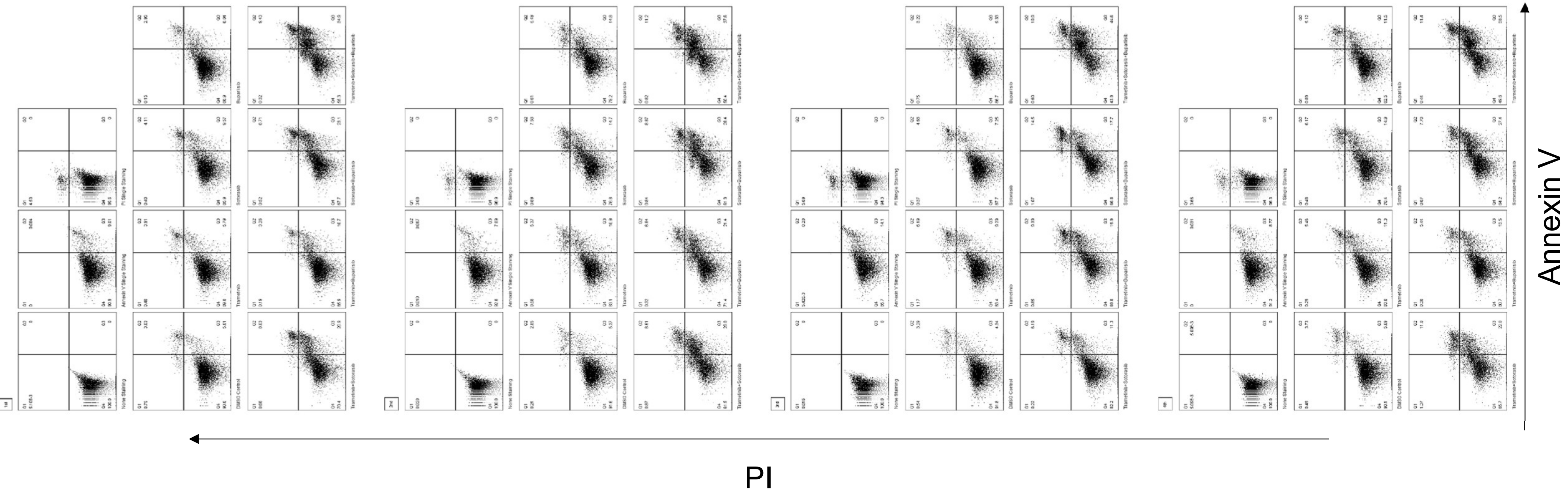

Supplementary Figure S4-5. Apoptosis/necrosis dot plot of MIA PACA-2 after 72 hours Sotorasib, Trametinib, Buparlisib and inhibitor combination exposure.
Q4: Annexin V-/PI-, viable cells
Q3: Annexin V+/PI-, early apoptotic cells
Q2: Annexin V+/PI+, late apoptotic/necrotic cells
Cell death = Q2+Q3

Supplementary Figure S5-1-1

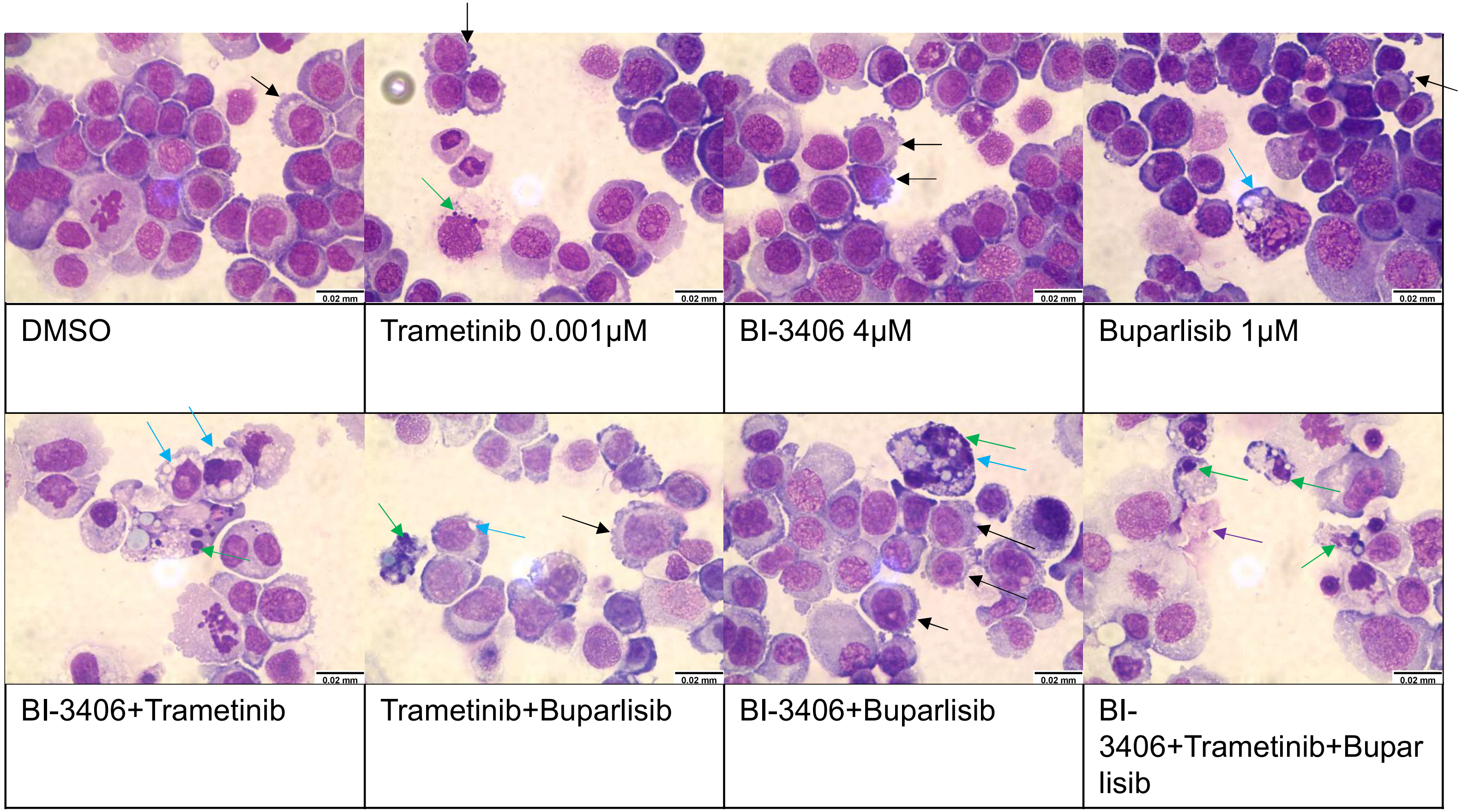

Supplementary Figure S5-1-1. Morphology changes of ASPC-1 after 72 hours BI-3406, Trametinib, Buparlisib and inhibitor combination exposure (1st). Magnification: 100×

↑ Membrane Bubbles, Membrane Bound Apoptotic Body, ↑ Vacuolization, ↑ Apoptotic Body, ↑ Nuclear Condensation/Fragmentation, ↑ Rupture of the Plasma Membrane

Supplementary Figure S5-1-2

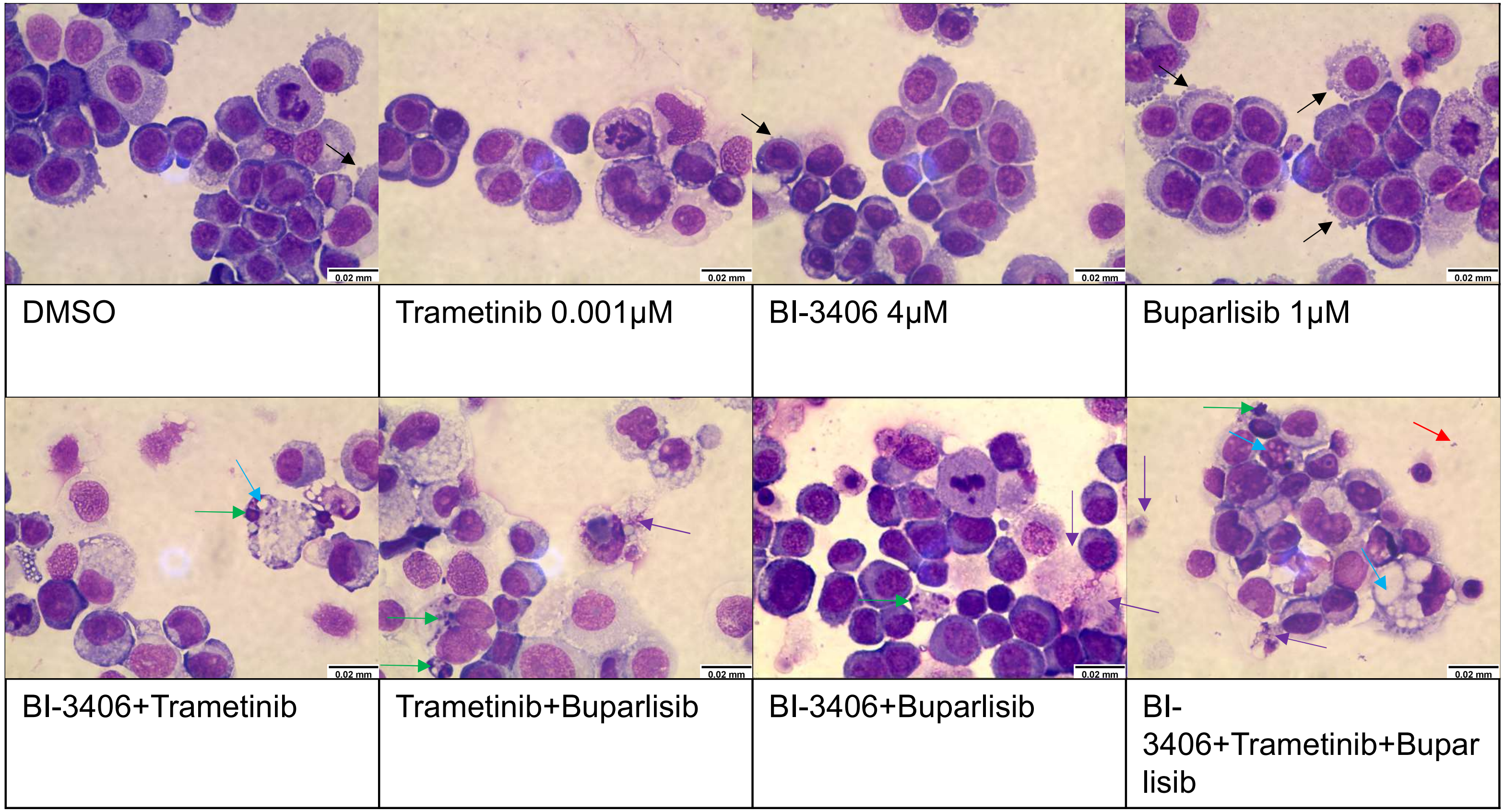

Supplementary Figure S5-1-2. Morphology changes of ASPC-1 after 72 hours BI-3406, Trametinib, Buparlisib and inhibitor combination exposure (2nd). Magnification: 100×

↑ Membrane Bubbles, Membrane Bound Apoptotic Body, ↑ Vacuolization, ↑ Apoptotic Body, ↑ Nuclear Condensation/Fragmentation, ↑ Rupture of the Plasma Membrane

Supplementary Figure S5-1-3

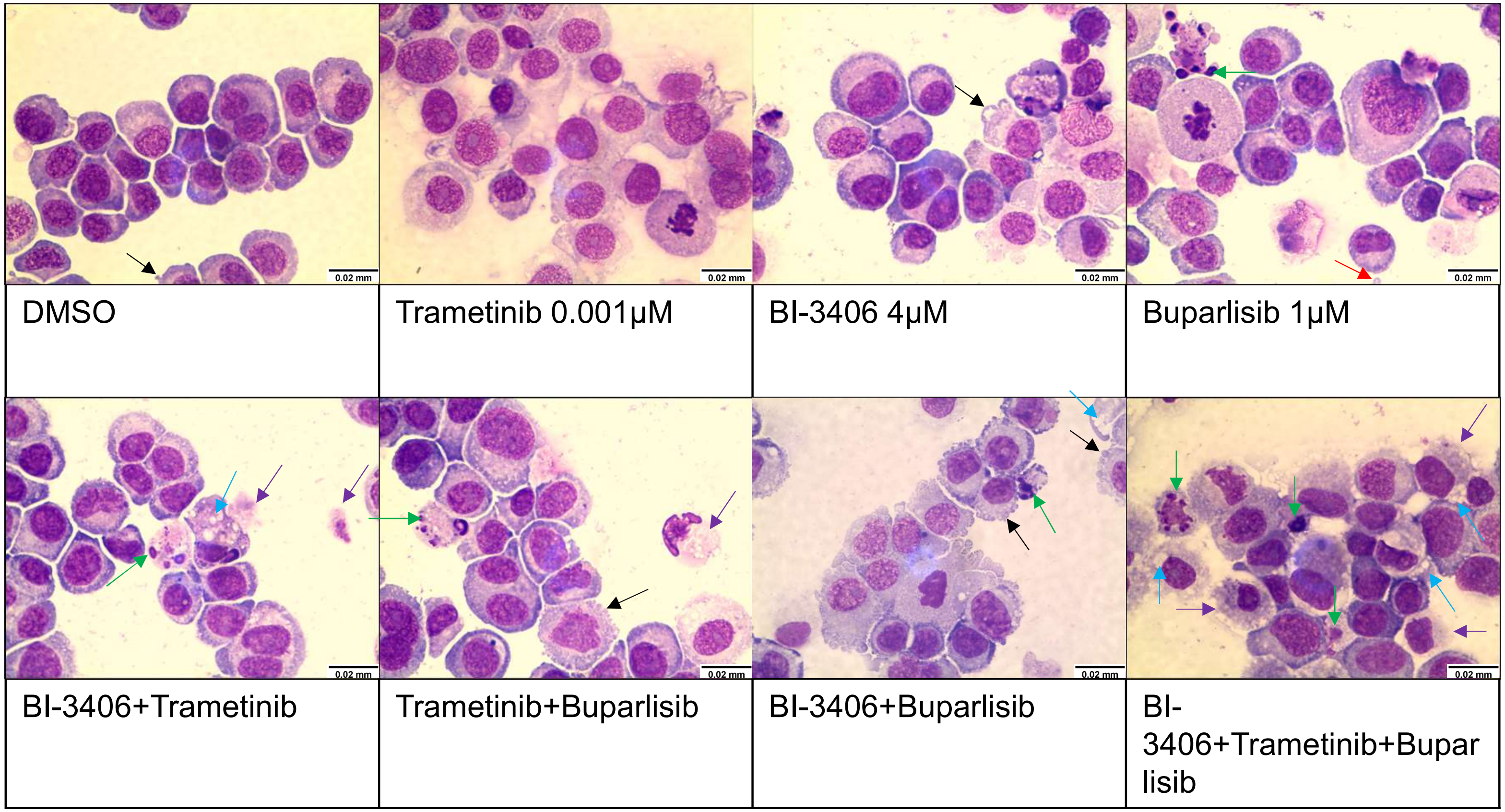

Supplementary Figure S5-1-3. Morphology changes of ASPC-1 after 72 hours BI-3406, Trametinib, Buparlisib and inhibitor combination exposure (3rd). Magnificent: 100×

↑ Membrane Bubbles, Membrane Bound Apoptotic Body, ↑ Vacuolization, ↑ Apoptotic Body, ↑ Nuclear Condensation/Fragmentation, ↑ Rupture of the Plasma Membrane

Supplementary Figure S5-1-4

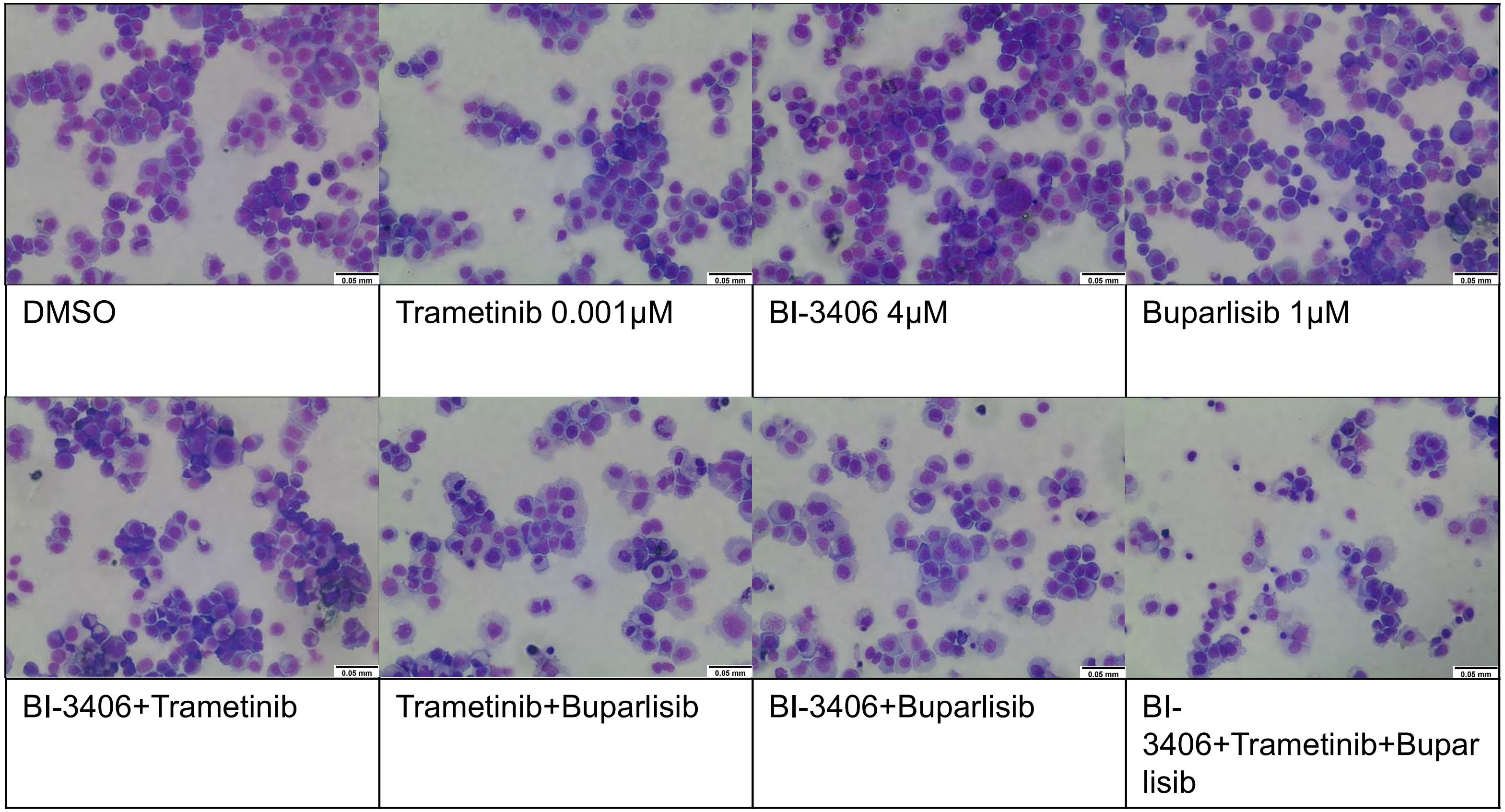

Supplementary Figure S5-1-4. Morphology changes of ASPC-1 after 72 hours BI-3406, Trametinib, Buparlisib and inhibitor combination exposure (1st). Magnification: 40×

Supplementary Figure S5-1-5

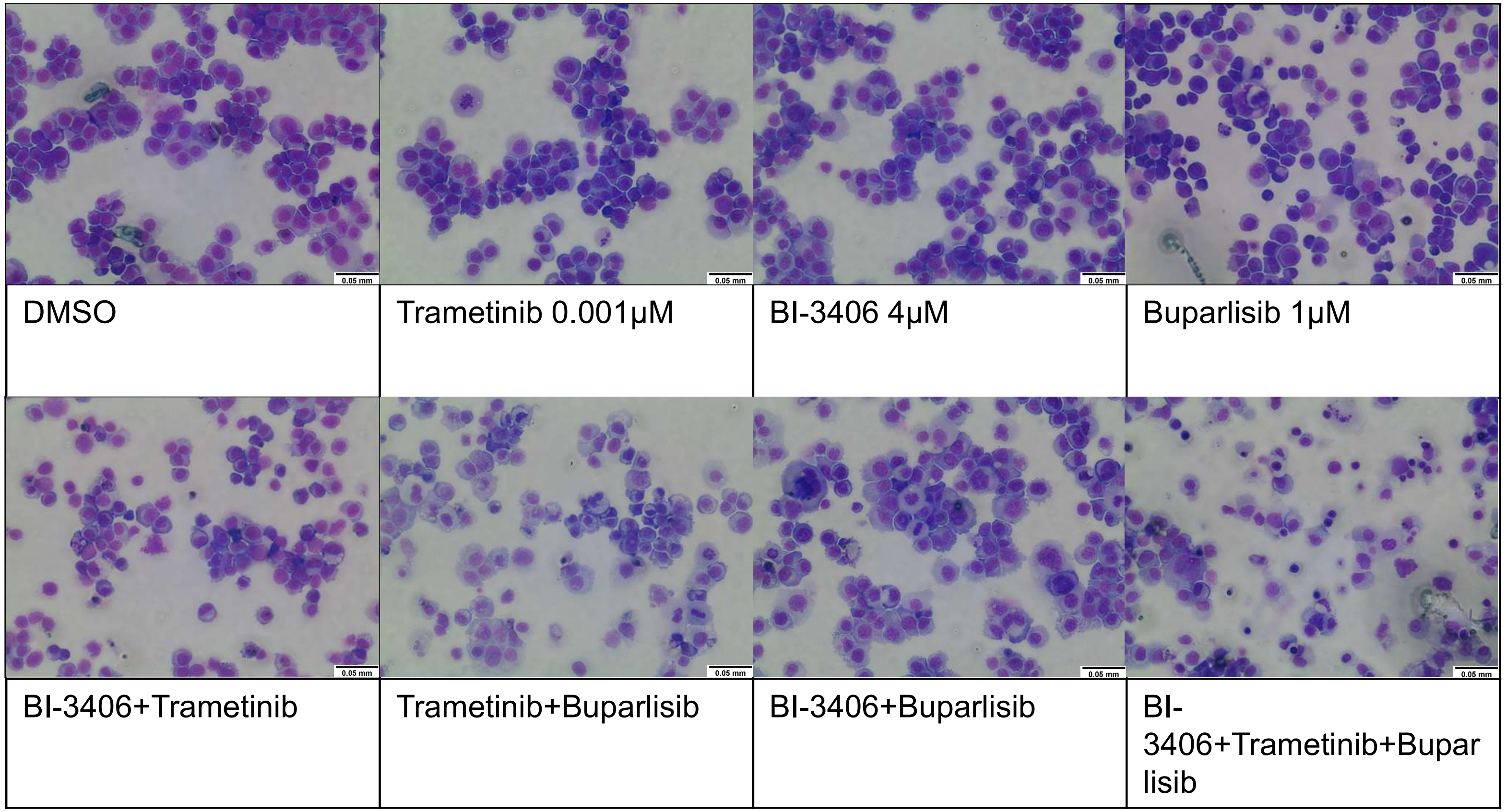

Supplementary Figure S5-1-5. Morphology changes of ASPC-1 after 72 hours BI-3406, Trametinib, Buparlisib and inhibitor combination exposure (2nd). Magnificent: 40×

Supplementary Figure S5-1-6

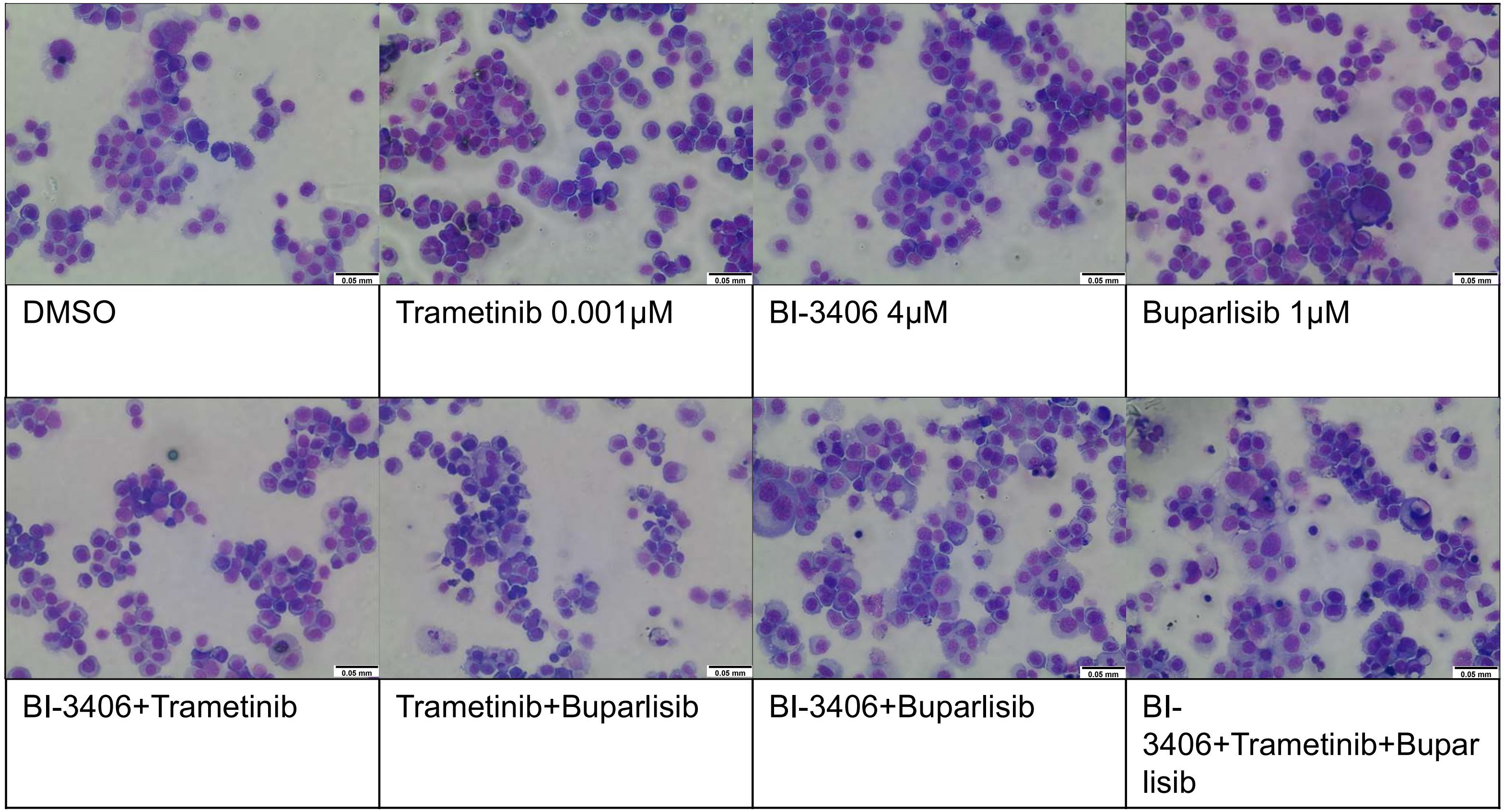

Supplementary Figure S5-1-6. Morphology changes of ASPC-1 after 72 hours BI-3406, Trametinib, Buparlisib and inhibitor combination exposure (3rd). Magnification: 40×

Supplementary Figure S5-2-1

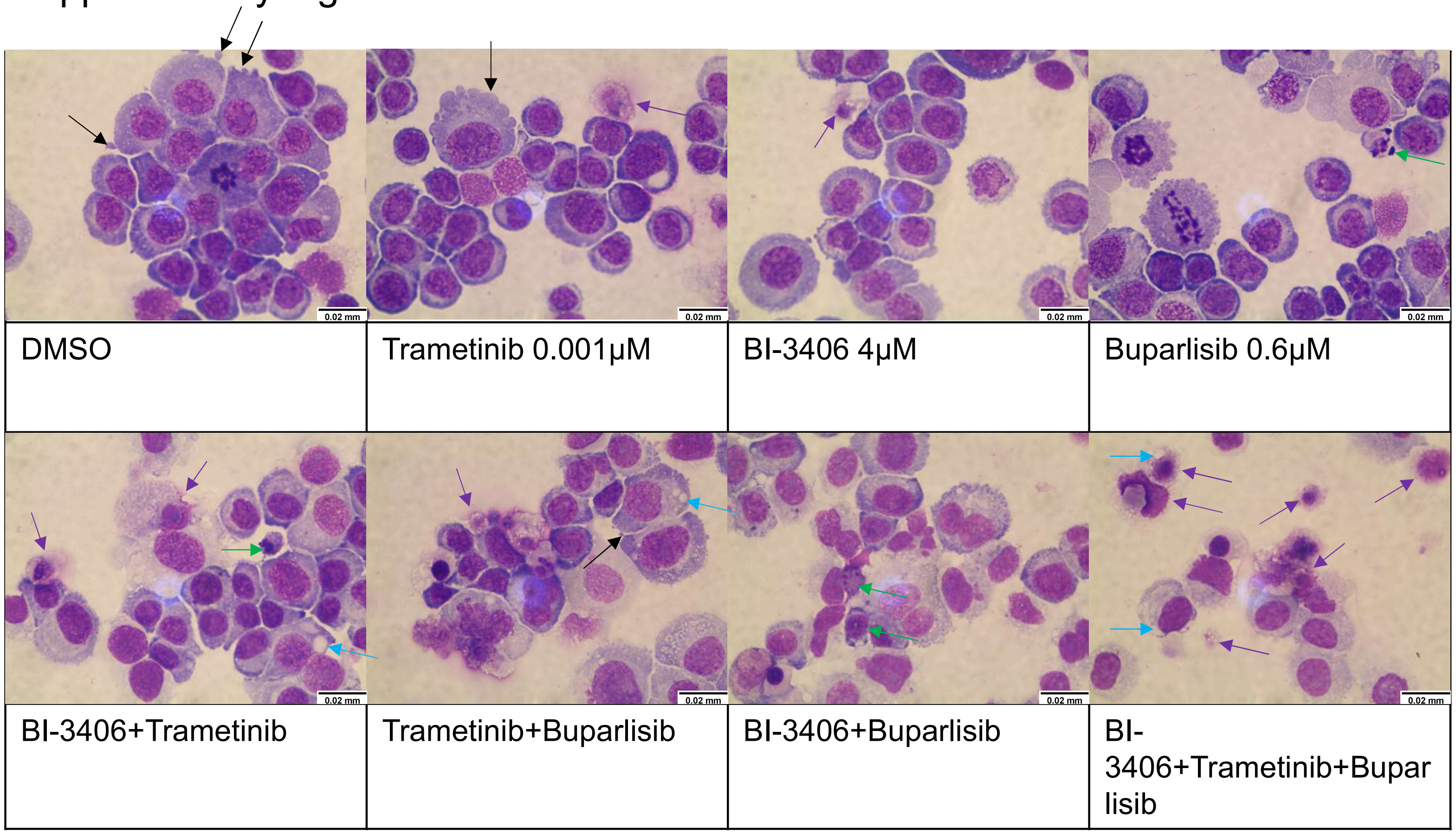

Supplementary Figure S5-2-1. Morphology changes of BXPC-3 after 72 hours BI-3406, Trametinib, Buparlisib and inhibitor combination exposure (1st). Magnification: 100×

↑ Membrane Bubbles, Membrane Bound Apoptotic Body, ↑ Vacuolization, ↑ Apoptotic Body, ↑ Nuclear Condensation/Fragmentation, ↑ Rupture of the Plasma Membrane

Supplementary Figure S5-2-2

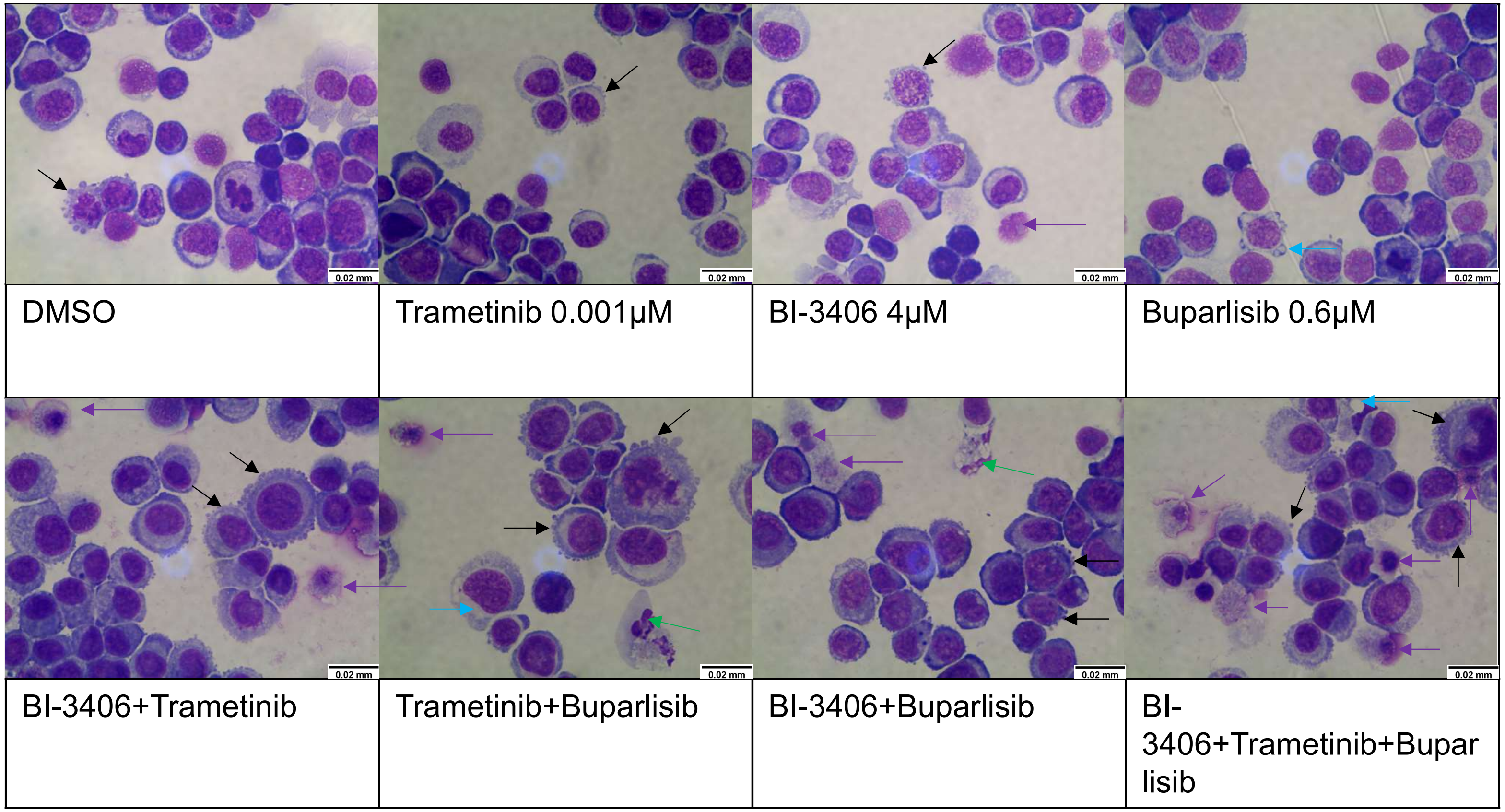

Supplementary Figure S5-2-2. Morphology changes of BXPC-3 after 72 hours BI-3406, Trametinib, Buparlisib and inhibitor combination exposure (2nd). Magnification: 100×

↑ Membrane Bubbles, Membrane Bound Apoptotic Body, ↑ Vacuolization, ↑ Apoptotic Body, ↑ Nuclear Condensation/Fragmentation, ↑ Rupture of the Plasma Membrane

Supplementary Figure S5-2-3

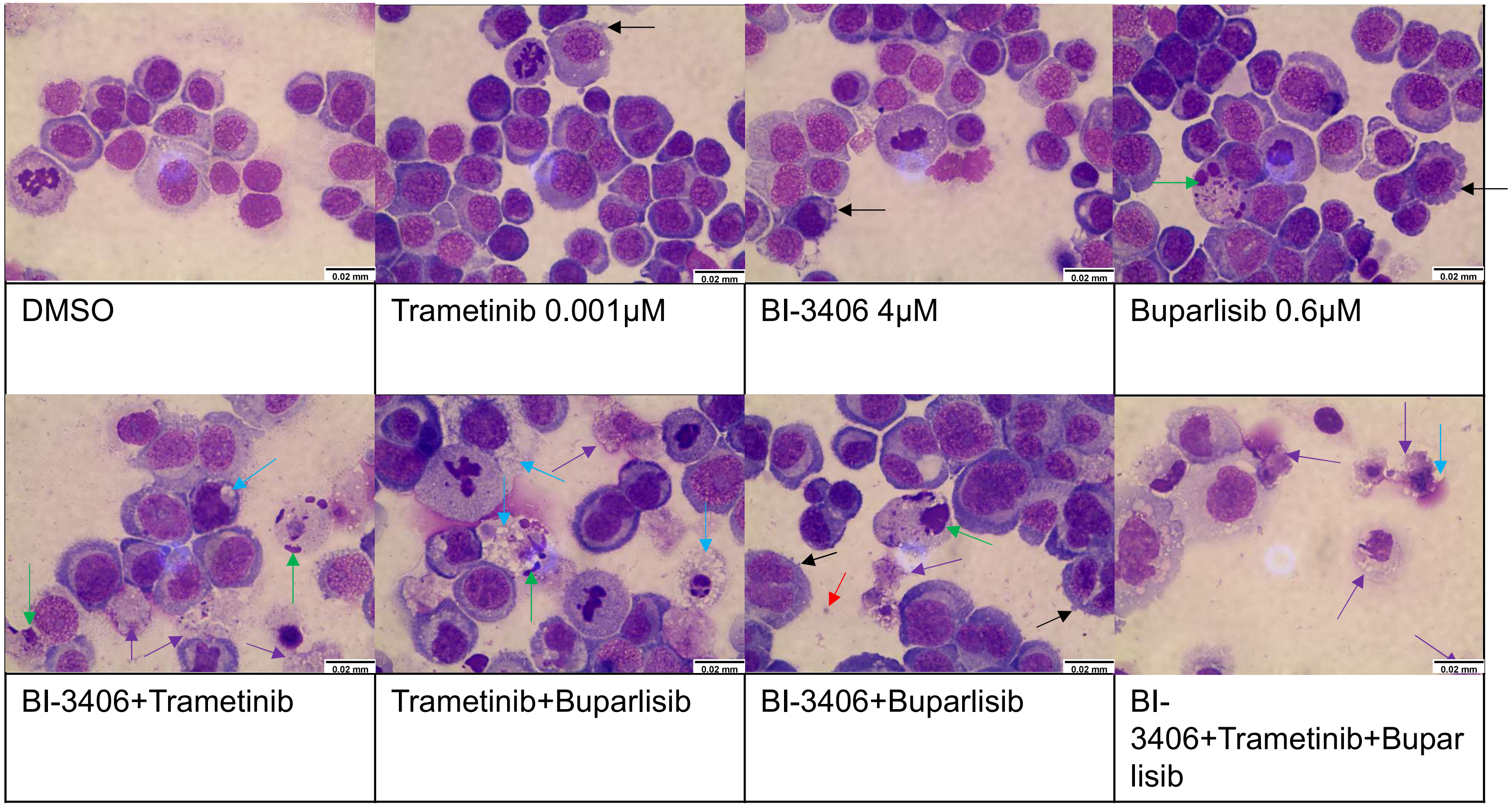

Supplementary Figure S5-2-3. Morphology changes of BXPC-3 after 72 hours BI-3406, Trametinib, Buparlisib and inhibitor combination exposure (3rd). Magnification: 100×

↑ Membrane Bubbles, Membrane Bound Apoptotic Body, ↑ Vacuolization, ↑ Apoptotic Body, ↑ Nuclear Condensation/Fragmentation, ↑ Rupture of the Plasma Membrane

Supplementary Figure S5-2-4

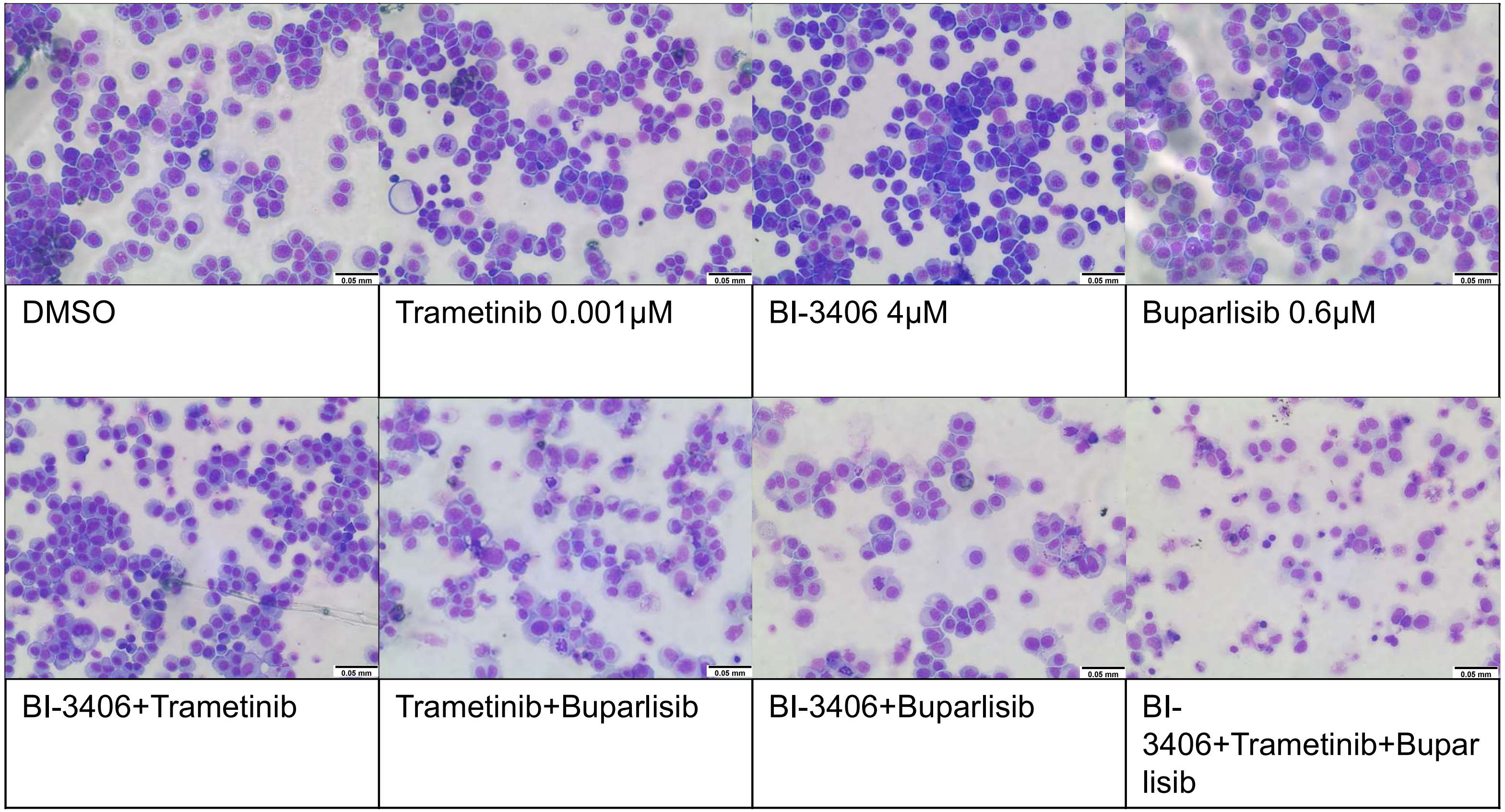

Supplementary Figure S5-2-4. Morphology changes of BXPC-3 after 72 hours BI-3406, Trametinib, Buparlisib and inhibitor combination exposure (1st). Magnification: 40×

Supplementary Figure S5-2-5

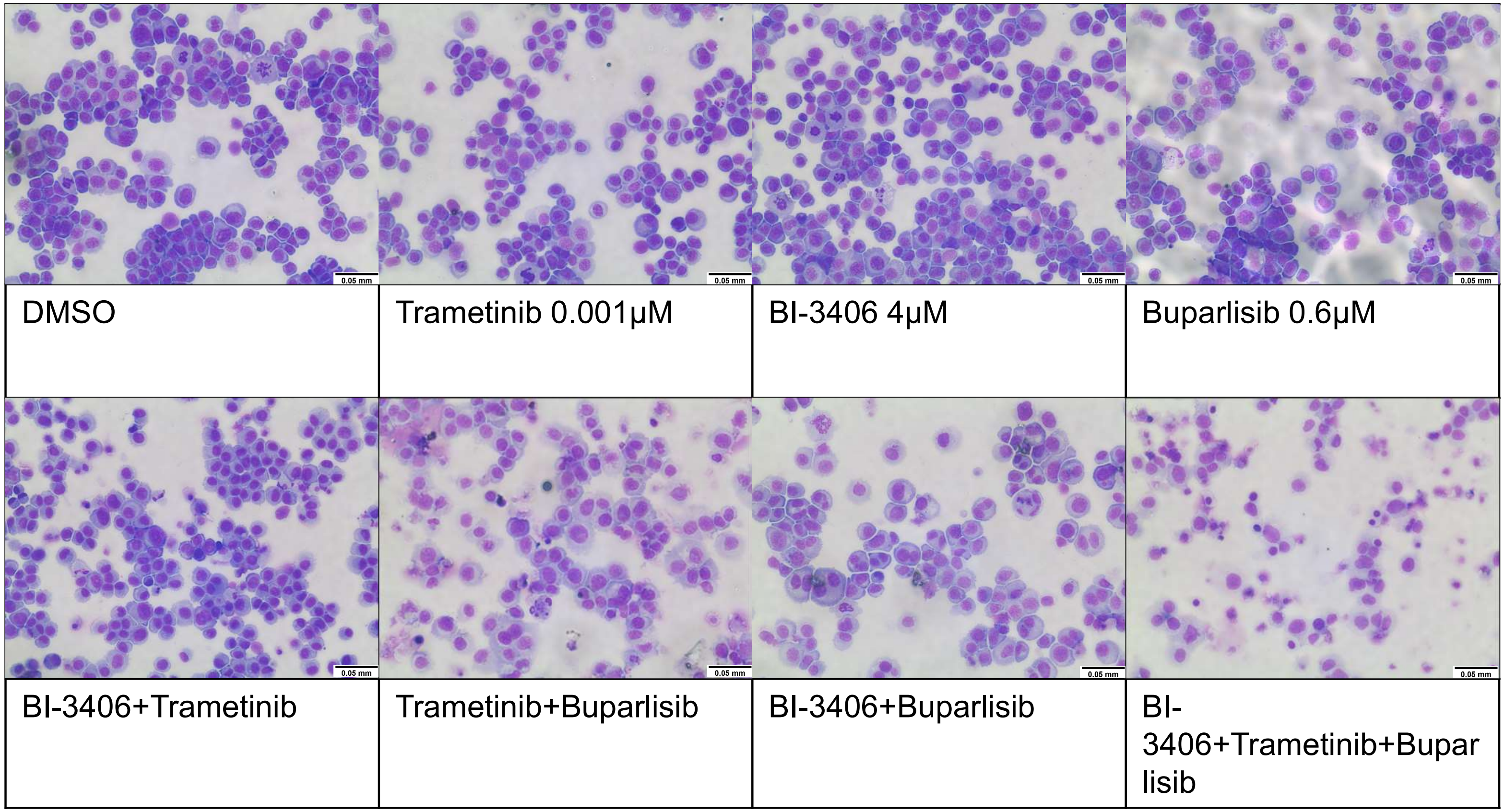

Supplementary Figure S5-2-5. Morphology changes of BXPC-3 after 72 hours BI-3406, Trametinib, Buparlisib and inhibitor combination exposure (2nd). Magnification: 40×

Supplementary Figure S5-2-6

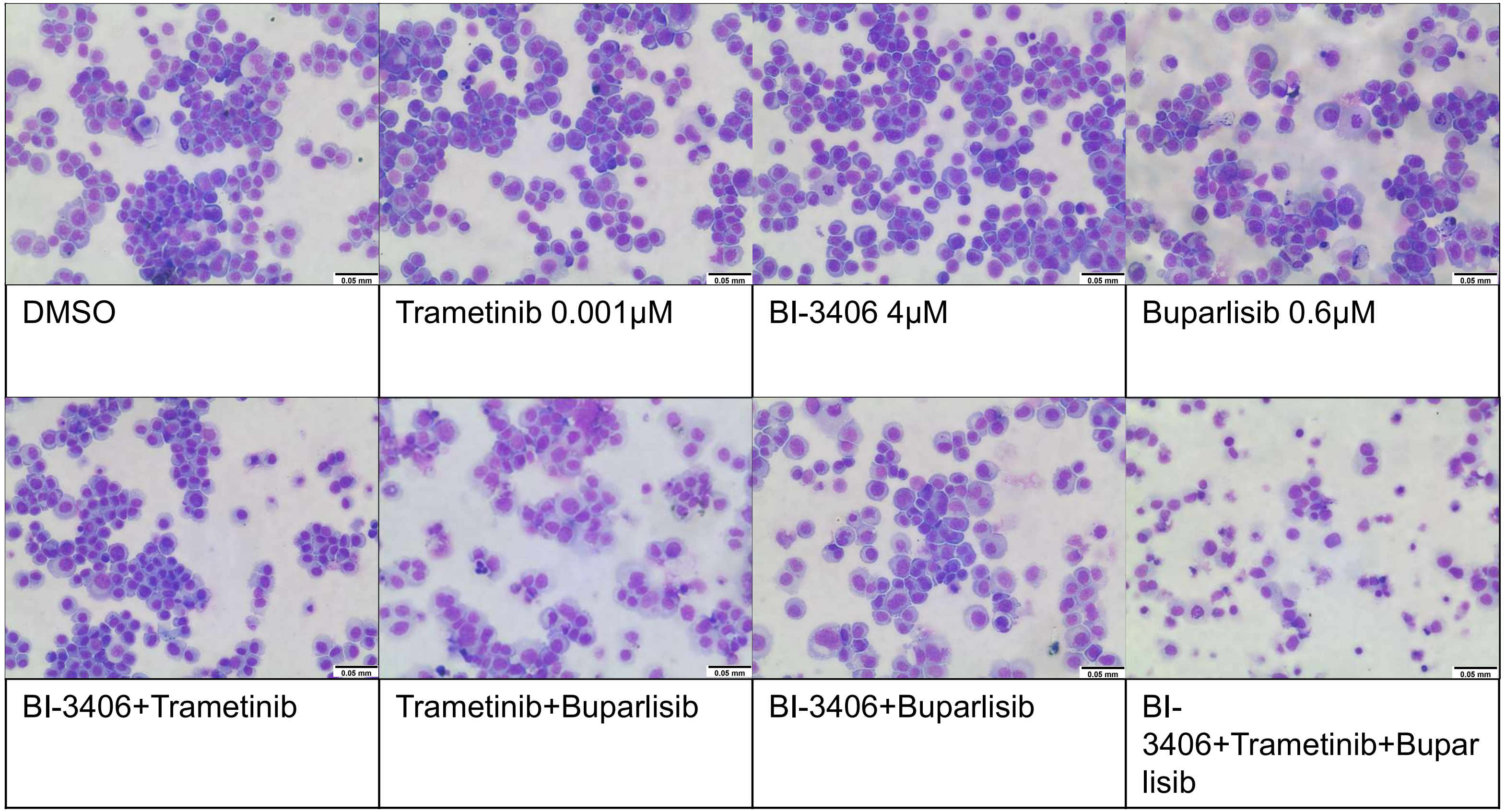

Supplementary Figure S5-2-6. Morphology changes of BXPC-3 after 72 hours BI-3406, Trametinib, Buparlisib and inhibitor combination exposure (3rd). Magnification: 40×

Supplementary Figure S5-3-1

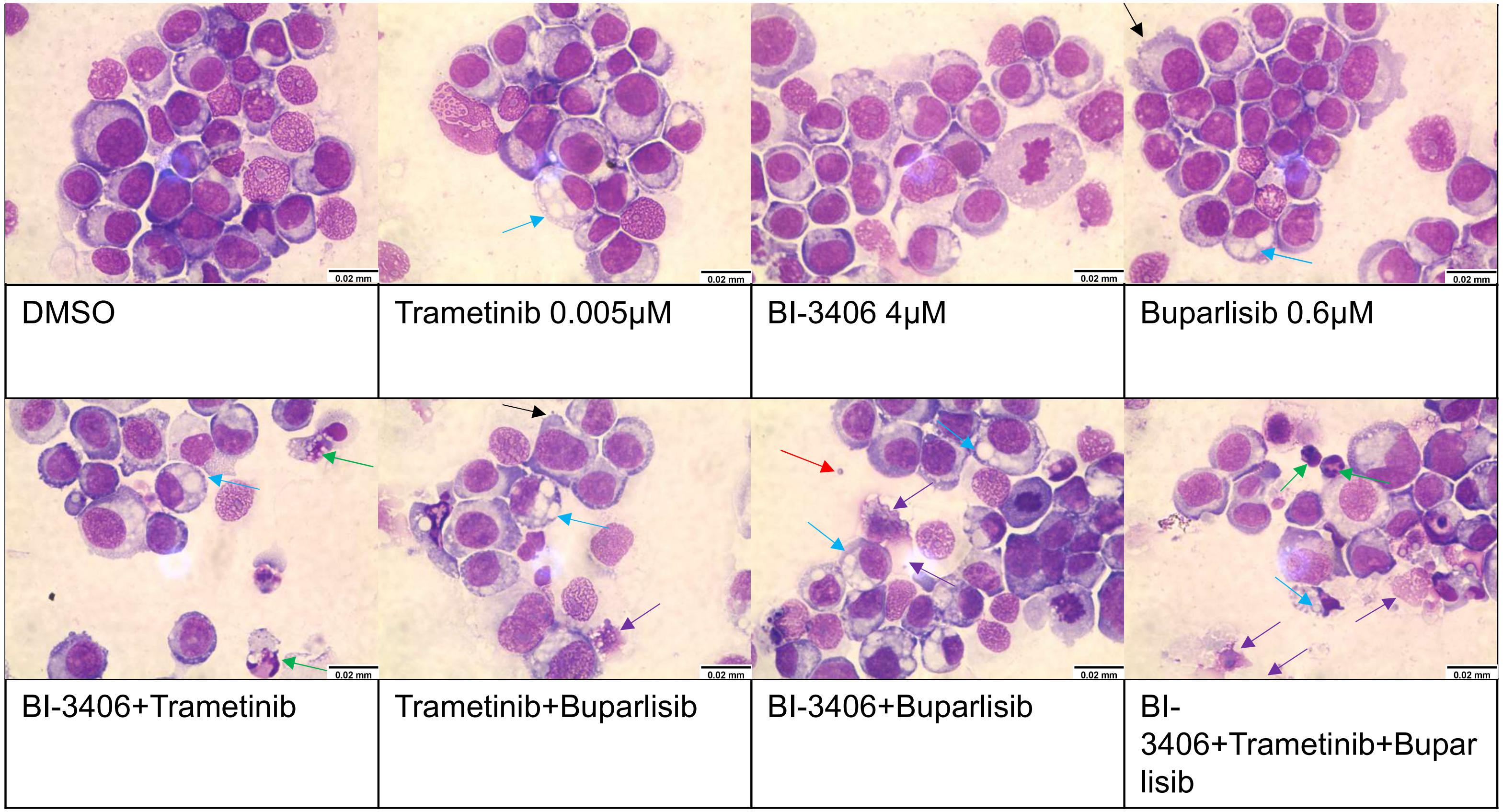

Supplementary Figure S5-3-1. Morphology changes of CAPAN-1 after 72 hours BI-3406, Trametinib, Buparlisib and inhibitor combination exposure (1st). Magnification: 100×

↑ Membrane Bubbles, Membrane Bound Apoptotic Body, ↑ Vacuolization, ↑ Apoptotic Body, ↑ Nuclear Condensation/Fragmentation, ↑ Rupture of the Plasma Membrane

Supplementary Figure S5-3-2

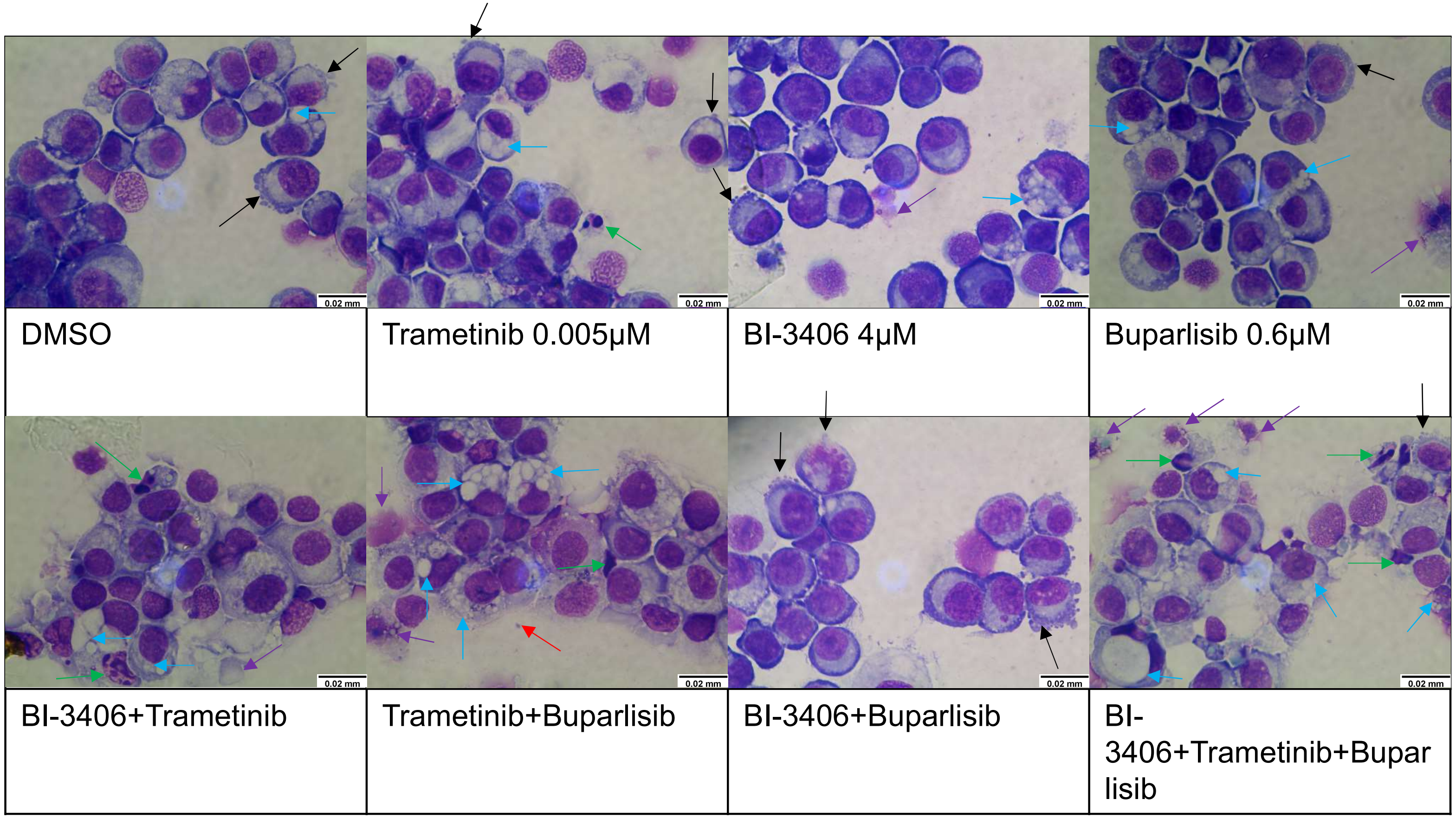

Supplementary Figure S5-3-2. Morphology changes of CAPAN-1 after 72 hours BI-3406, Trametinib, Buparlisib and inhibitor combination exposure (2nd). Magnification: 100×

↑ Membrane Bubbles, Membrane Bound Apoptotic Body, ↑ Vacuolization, ↑ Apoptotic Body, ↑ Nuclear Condensation/Fragmentation, ↑ Rupture of the Plasma Membrane

Supplementary Figure S5-3-3

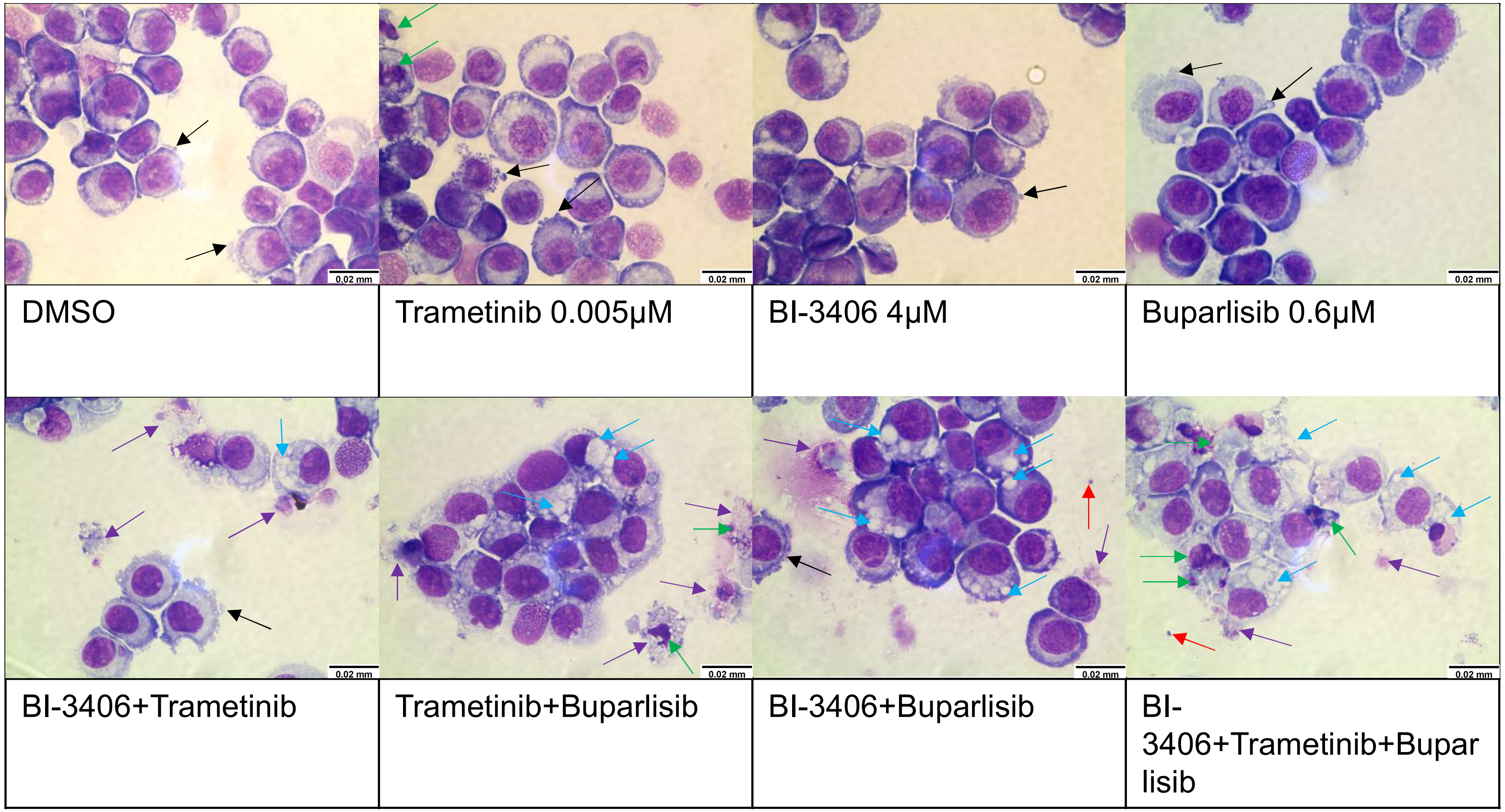

Supplementary Figure S5-3-3. Morphology changes of CAPAN-1 after 72 hours BI-3406, Trametinib, Buparlisib and inhibitor combination exposure (3rd). Magnification: 100×

↑ Membrane Bubbles, Membrane Bound Apoptotic Body, ↑ Vacuolization, ↑ Apoptotic Body, ↑ Nuclear Condensation/Fragmentation, ↑ Rupture of the Plasma Membrane

Supplementary Figure S5-3-4

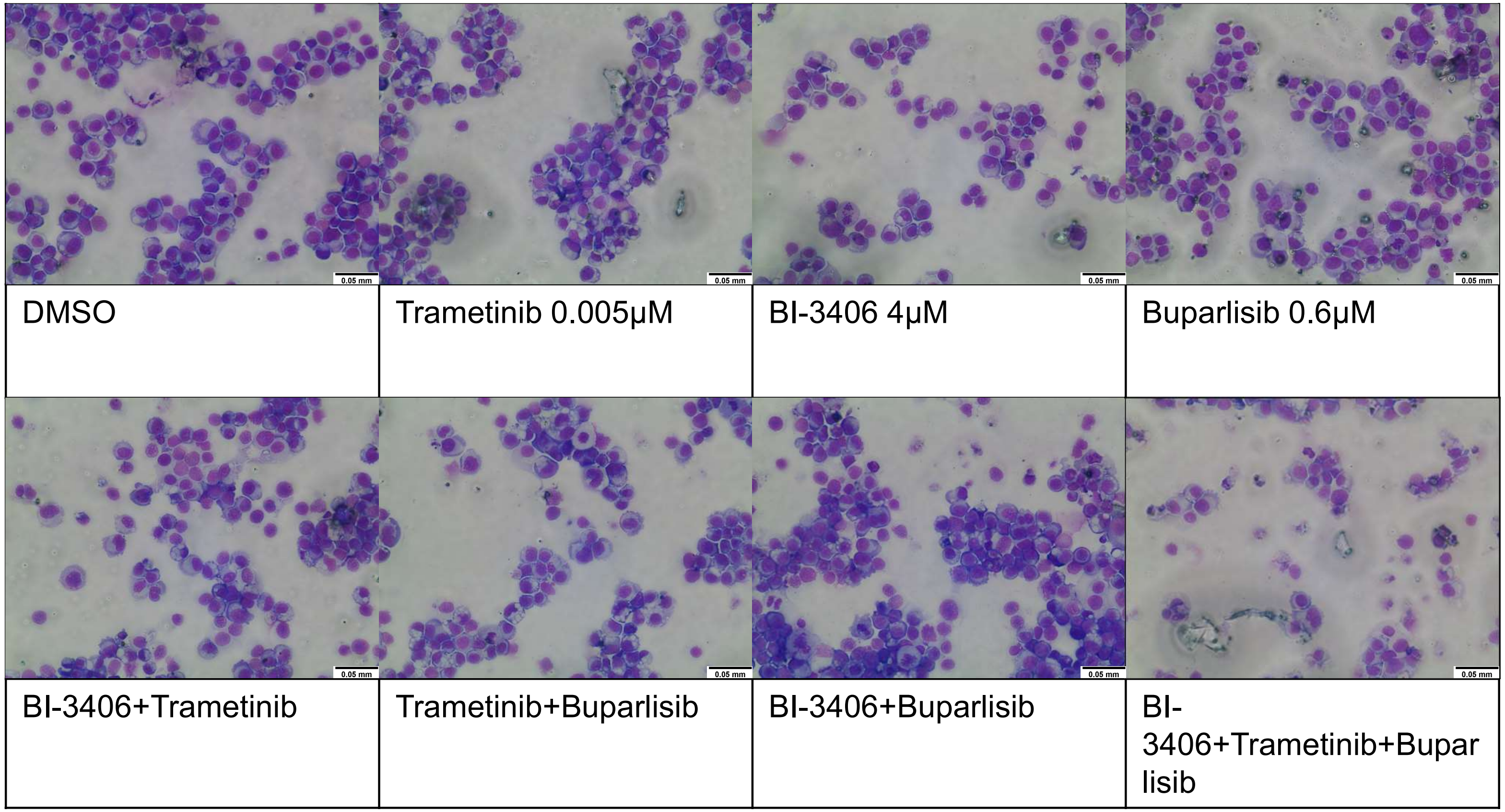

Supplementary Figure S5-3-4. Morphology changes of CAPAN-1 after 72 hours BI-3406, Trametinib, Buparlisib and inhibitor combination exposure (1st). Magnification: 40×

Supplementary Figure S5-3-5

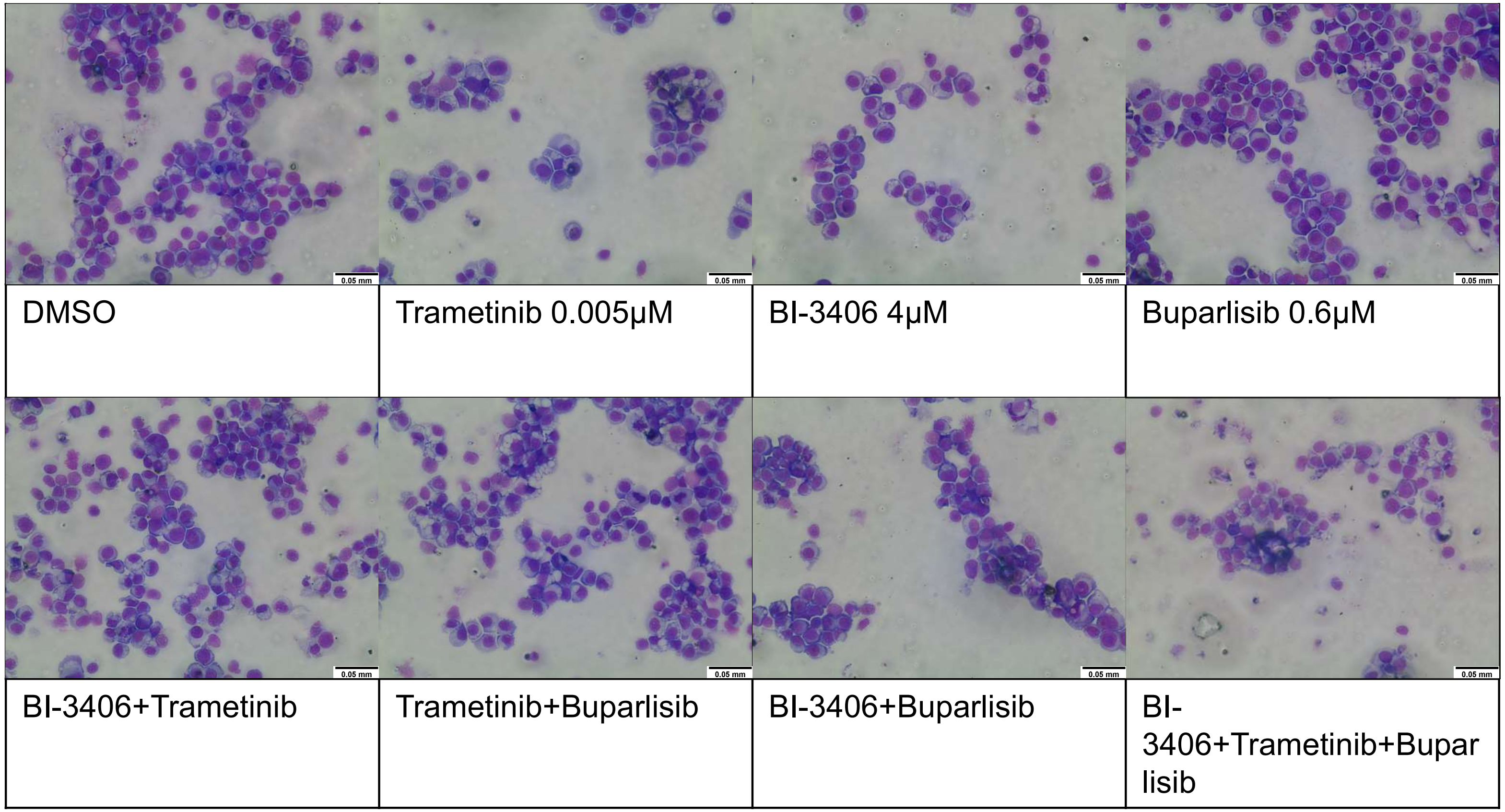

Supplementary Figure S5-3-5. Morphology changes of CAPAN-1 after 72 hours BI-3406, Trametinib, Buparlisib and inhibitor combination exposure (2nd). Magnification: 40×

Supplementary Figure S5-3-6

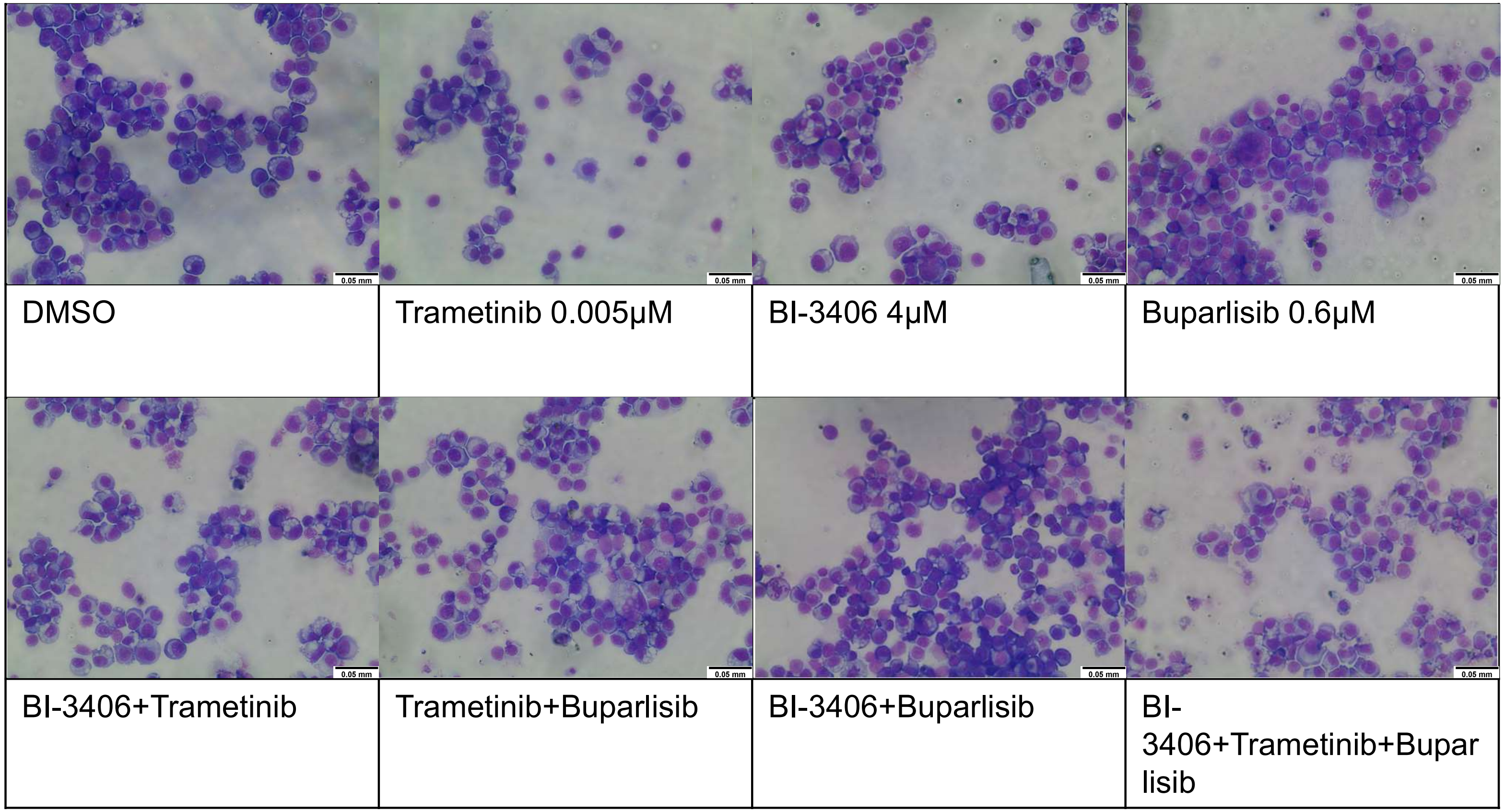

Supplementary Figure S5-3-6. Morphology changes of CAPAN-1 after 72 hours BI-3406, Trametinib, Buparlisib and inhibitor combination exposure (3rd). Magnification: 40×

Supplementary Figure S5-4-1

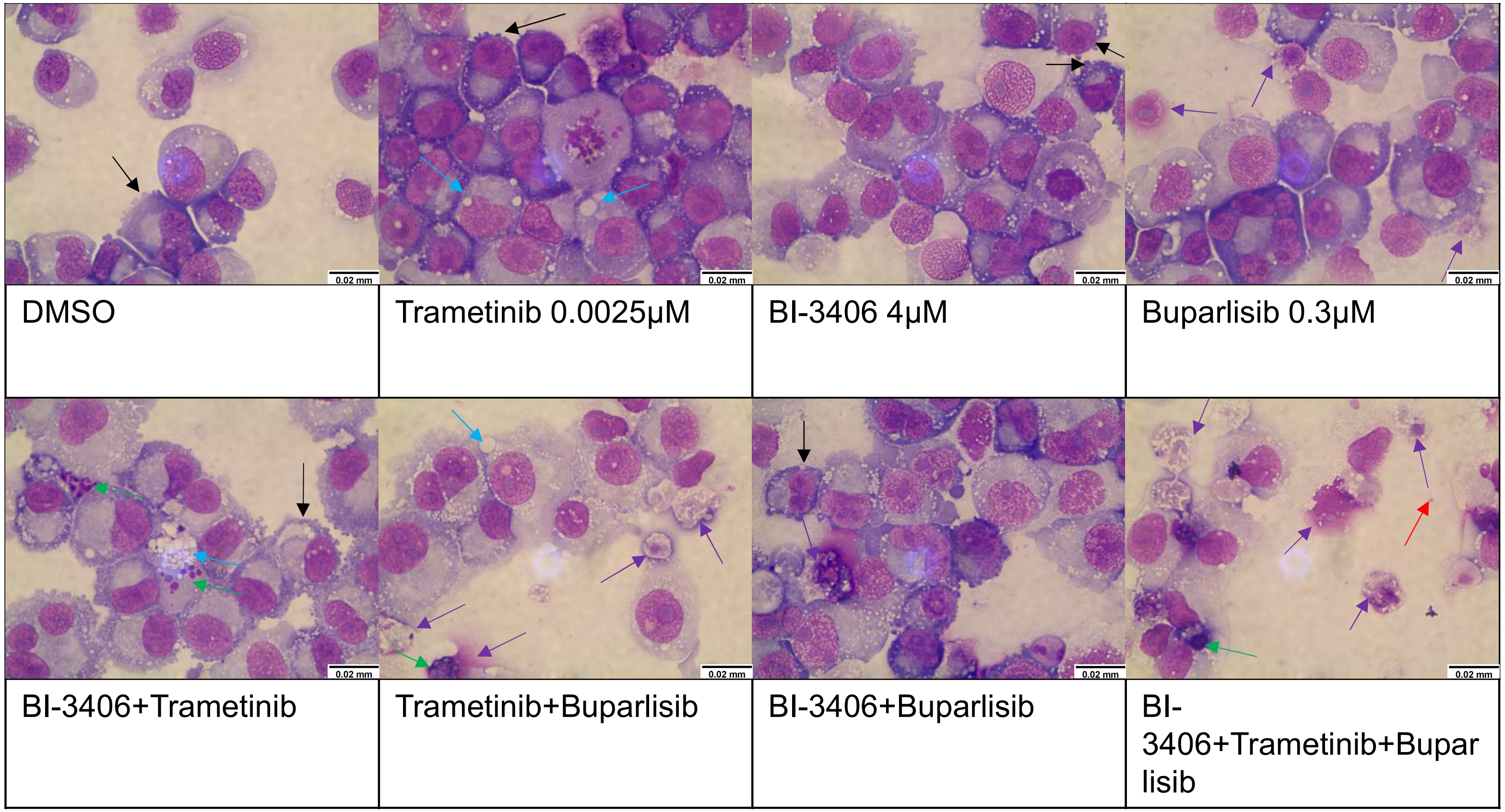

Supplementary Figure S5-4-1. Morphology changes of MIA PACA-2 after 72 hours BI-3406, Trametinib, Buparlisib and inhibitor combination exposure (1st). Magnification: 100×

↑ Membrane Bubbles, Membrane Bound Apoptotic Body, ↑ Vacuolization, ↑ Apoptotic Body, ↑ Nuclear Condensation/Fragmentation, ↑ Rupture of the Plasma Membrane

Supplementary Figure S5-4-2

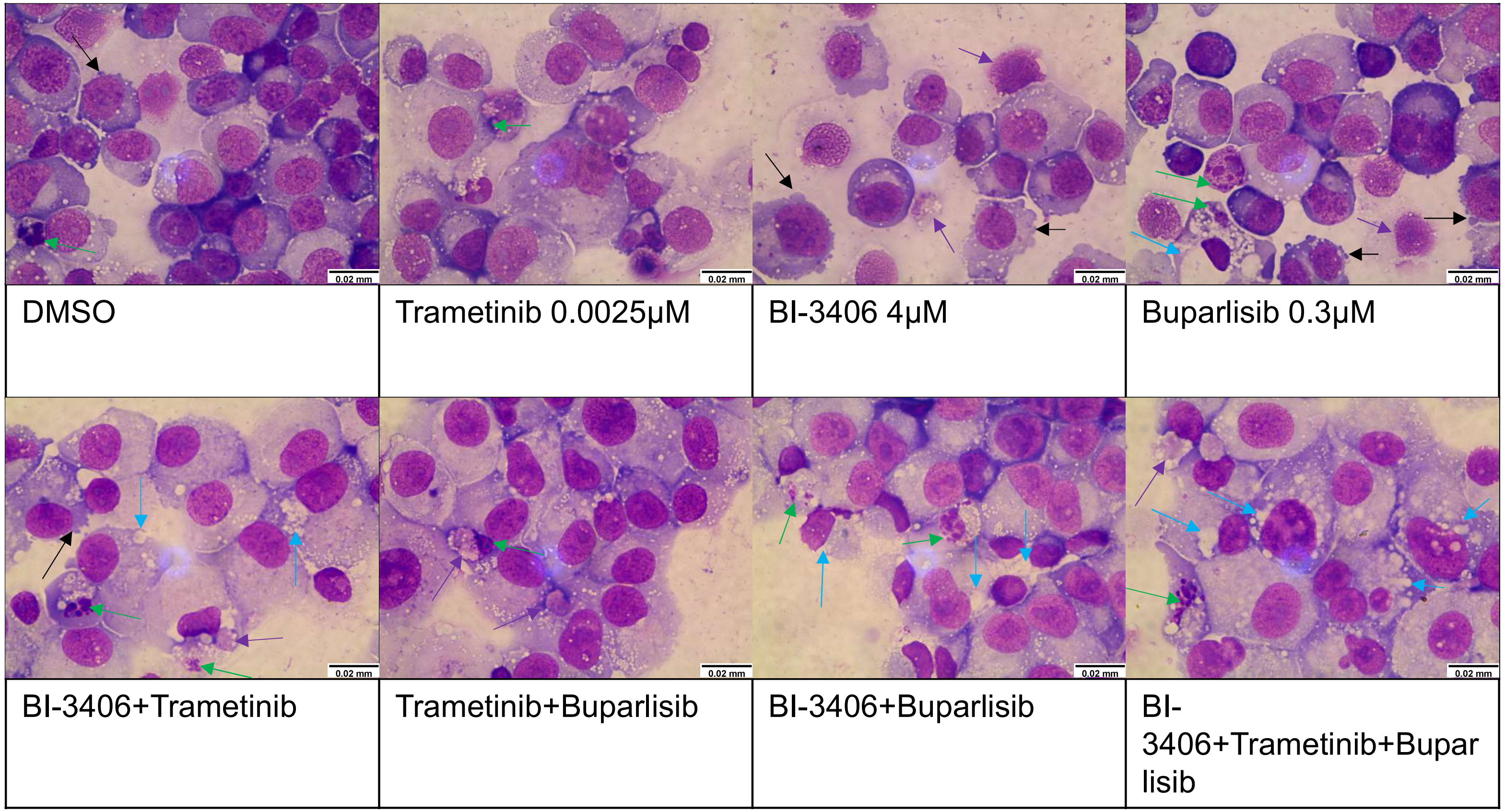

Supplementary Figure S5-4-2. Morphology changes of MIA PACA-2 after 72 hours BI-3406, Trametinib, Buparlisib and inhibitor combination exposure (2nd). Magnification: 100×

↑ Membrane Bubbles, Membrane Bound Apoptotic Body, ↑ Vacuolization, ↑ Apoptotic Body, ↑ Nuclear Condensation/Fragmentation, ↑ Rupture of the Plasma Membrane

Supplementary Figure S5-4-3

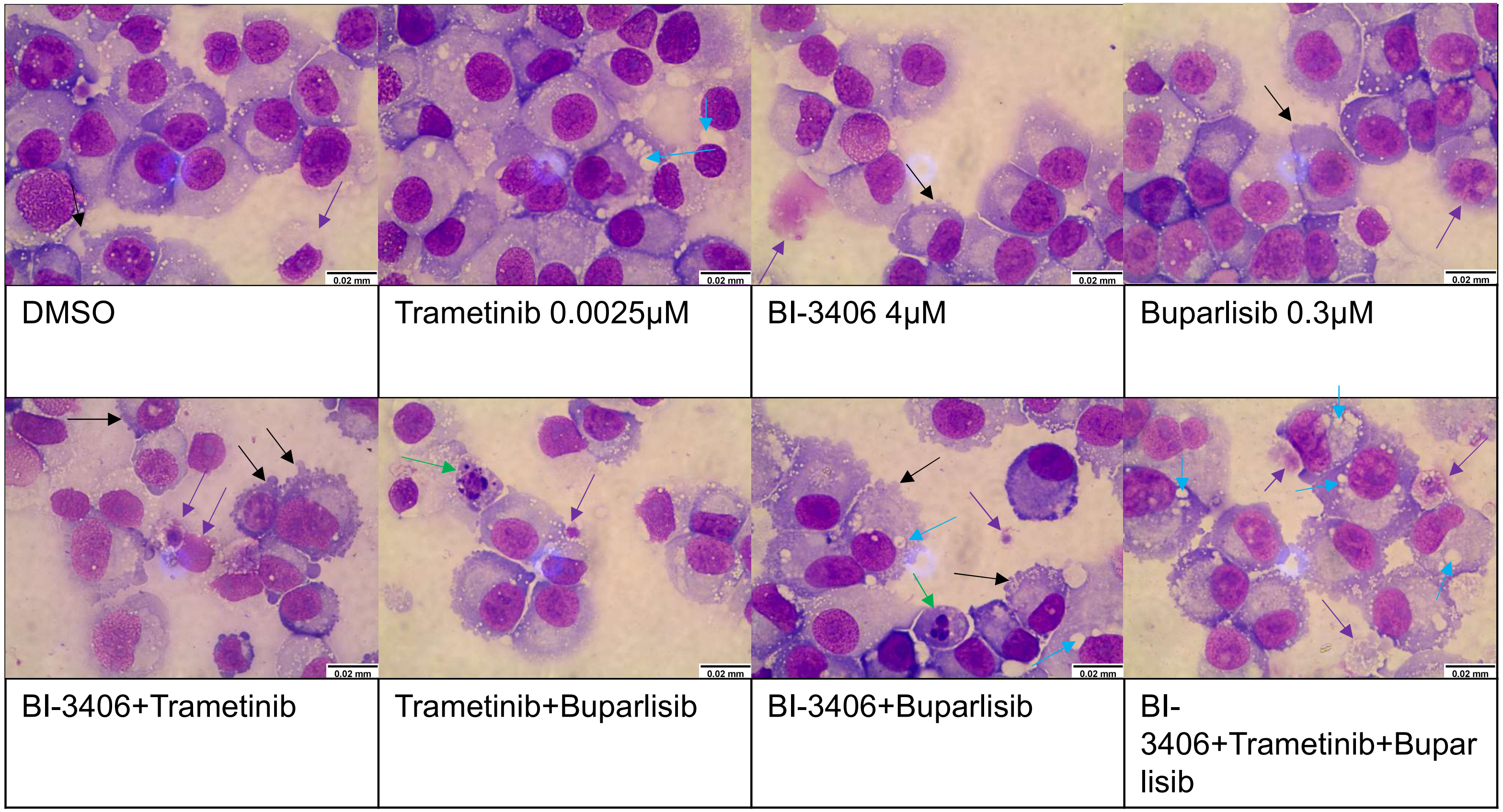

Supplementary Figure S5-4-3. Morphology changes of MIA PACA-2 after 72 hours BI-3406, Trametinib, Buparlisib and inhibitor combination exposure (3rd). Magnification: 100×

↑ Membrane Bubbles, Membrane Bound Apoptotic Body, ↑ Vacuolization, ↑ Apoptotic Body, ↑ Nuclear Condensation/Fragmentation, ↑ Rupture of the Plasma Membrane

Supplementary Figure S5-4-4

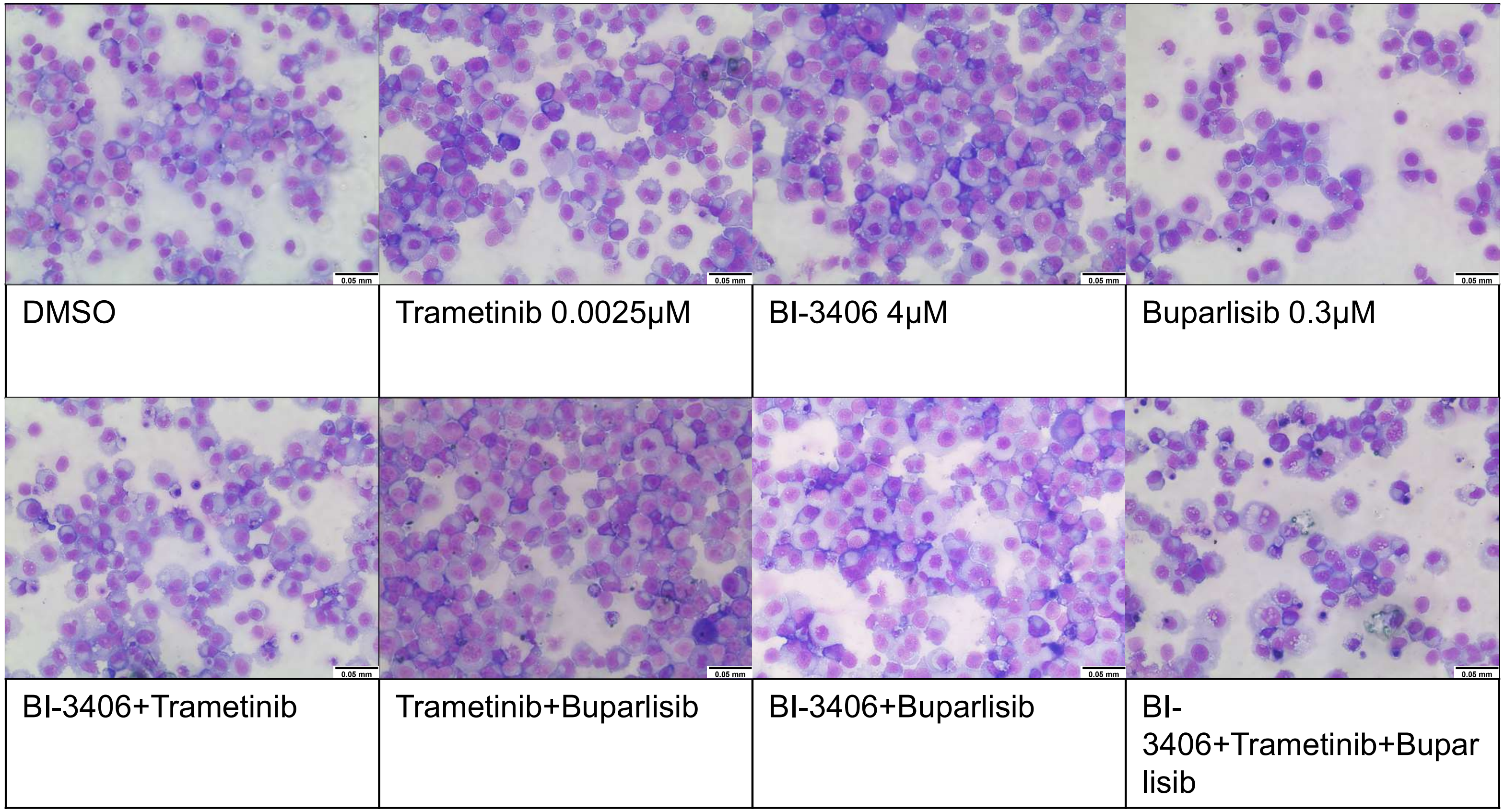

Supplementary Figure S5-4-4. Morphology changes of MIA PACA-2 after 72 hours BI-3406, Trametinib, Buparlisib and inhibitor combination exposure (1st). Magnification: 40×

Supplementary Figure S5-4-5

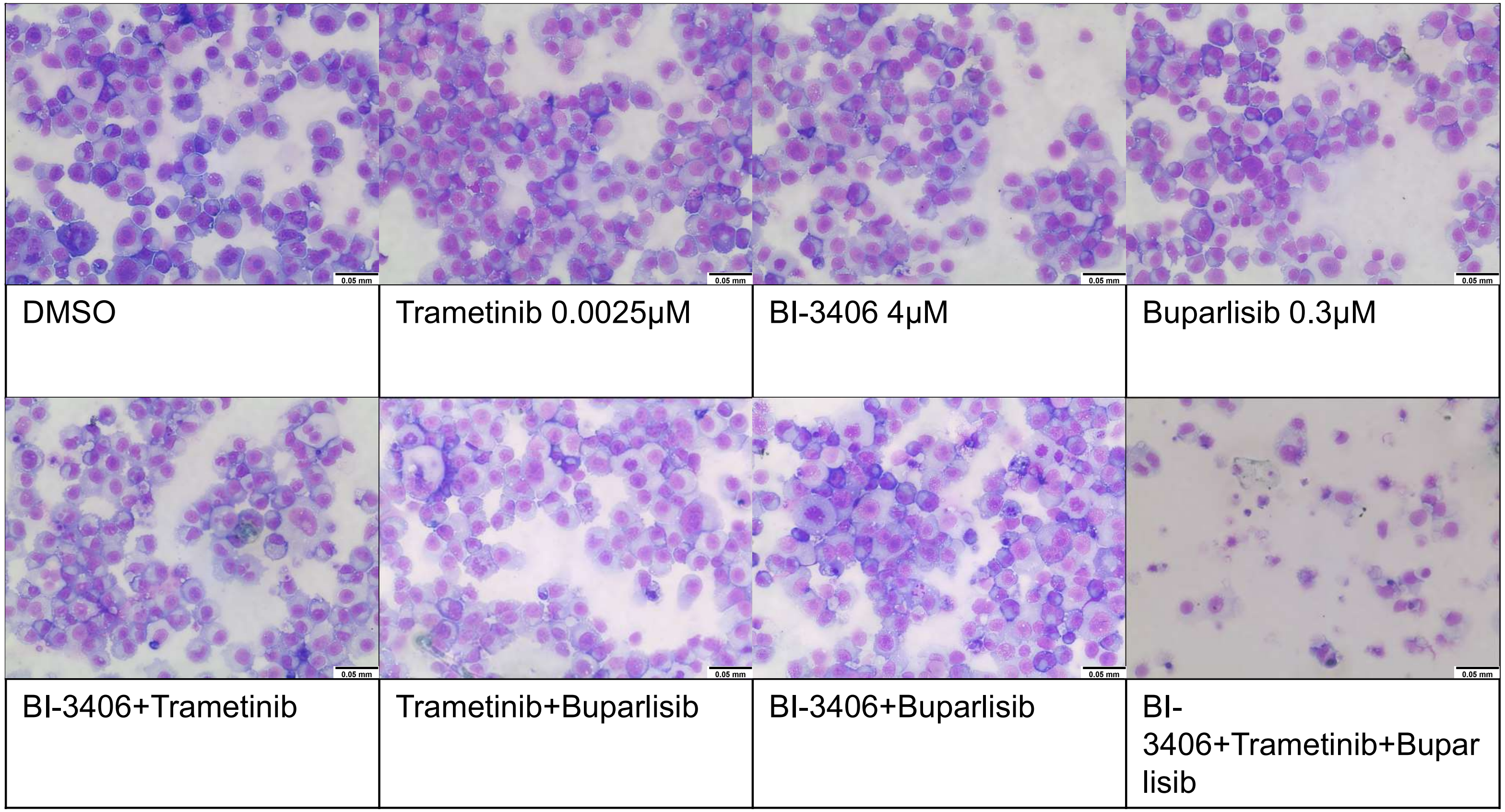

Supplementary Figure S5-4-5. Morphology changes of MIA PACA-2 after 72 hours BI-3406, Trametinib, Buparlisib and inhibitor combination exposure (2nd). Magnification: 40×

Supplementary Figure S5-4-6

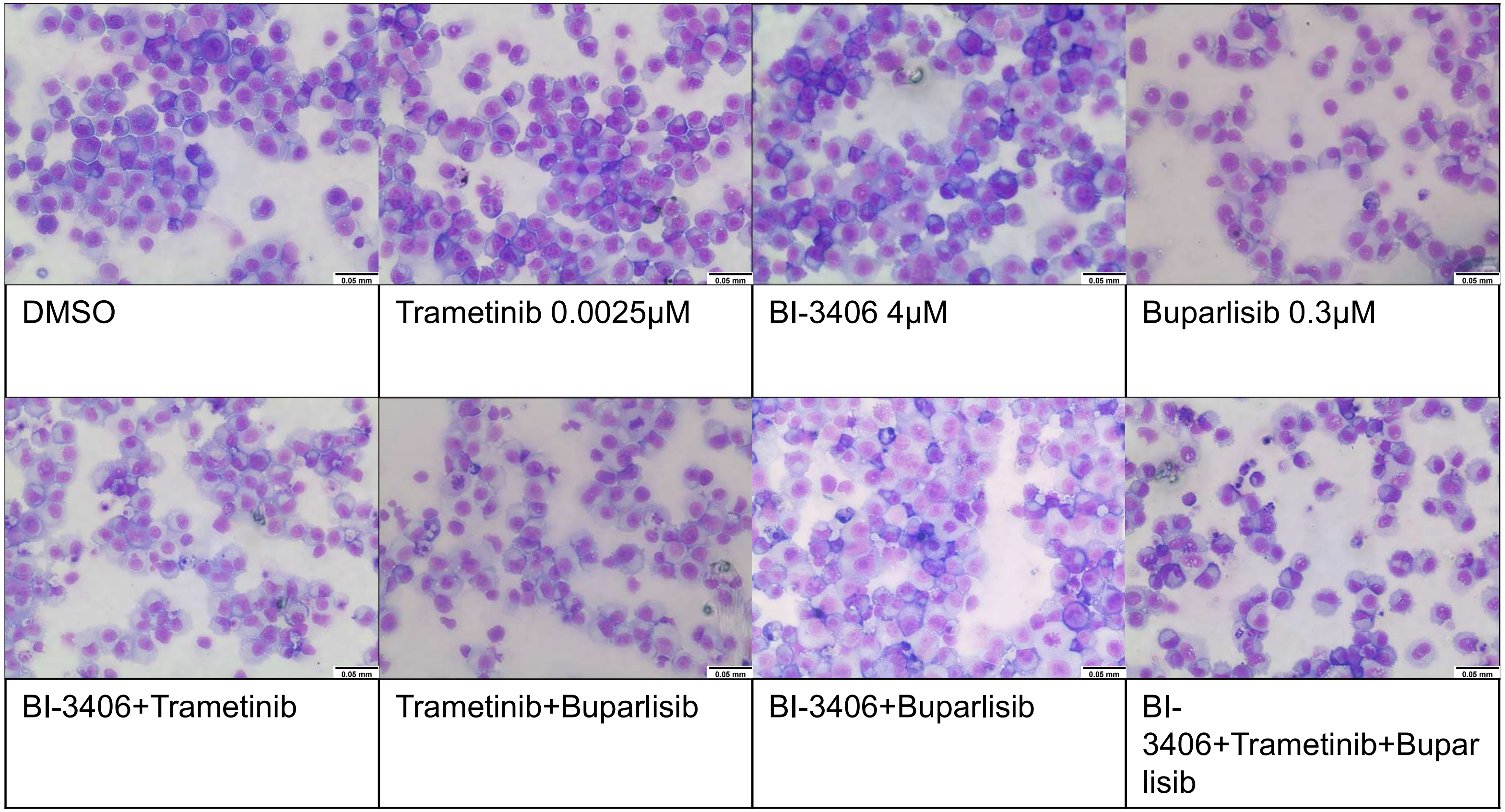

Supplementary Figure S5-4-6. Morphology changes of MIA PACA-2 after 72 hours BI-3406, Trametinib, Buparlisib and inhibitor combination exposure (3rd). Magnification: 40×

Supplementary Figure S5-5-1

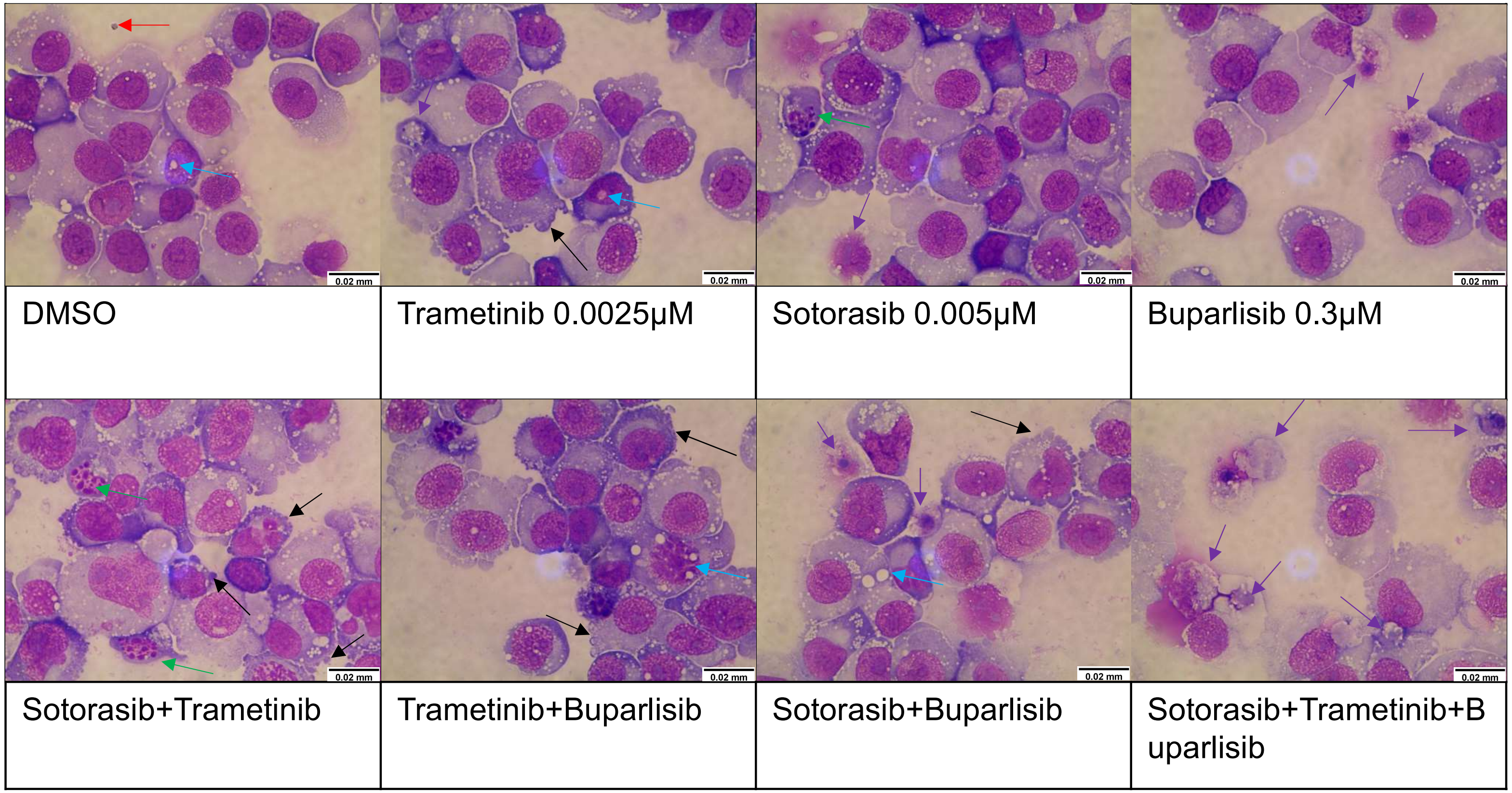

Supplementary Figure S5-5-1. Morphology changes of MIA PACA-2 after 72 hours Sotorasib, Trametinib, Buparlisib and inhibitor combination exposure (1st). Magnification: 100×

↑ Membrane Bubbles, Membrane Bound Apoptotic Body, ↑ Vacuolization, ↑ Apoptotic Body, ↑ Nuclear Condensation/Fragmentation, ↑ Rupture of the Plasma Membrane

Supplementary Figure S5-5-2

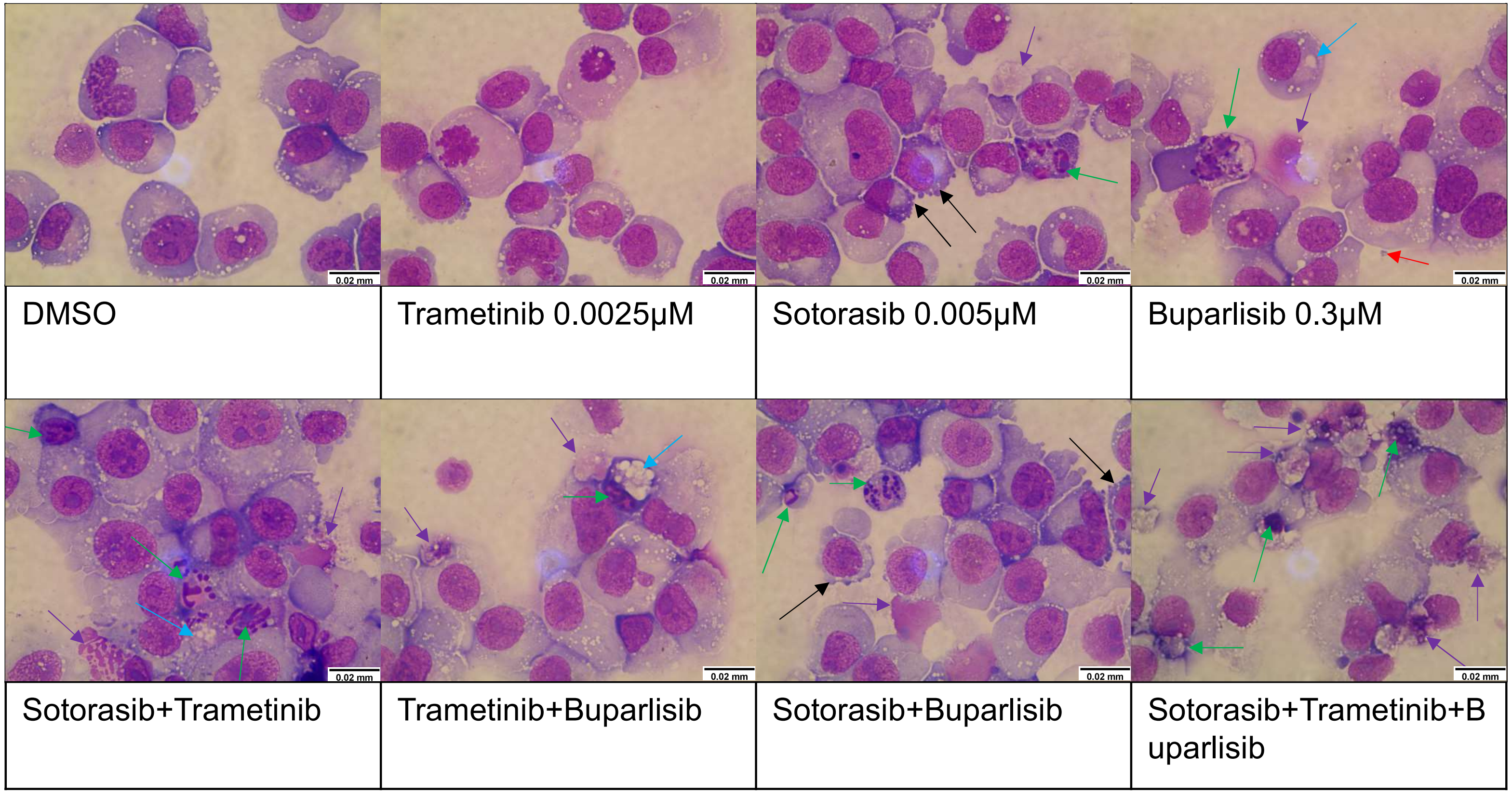

Supplementary Figure S5-5-2. Morphology changes of MIA PACA-2 after 72 hours Sotorasib, Trametinib, Buparlisib and inhibitor combination exposure (2nd). Magnification: 100×

↑ Membrane Bubbles, Membrane Bound Apoptotic Body, ↑ Vacuolization, ↑ Apoptotic Body, ↑ Nuclear Condensation/Fragmentation, ↑ Rupture of the Plasma Membrane

Supplementary Figure S5-5-3

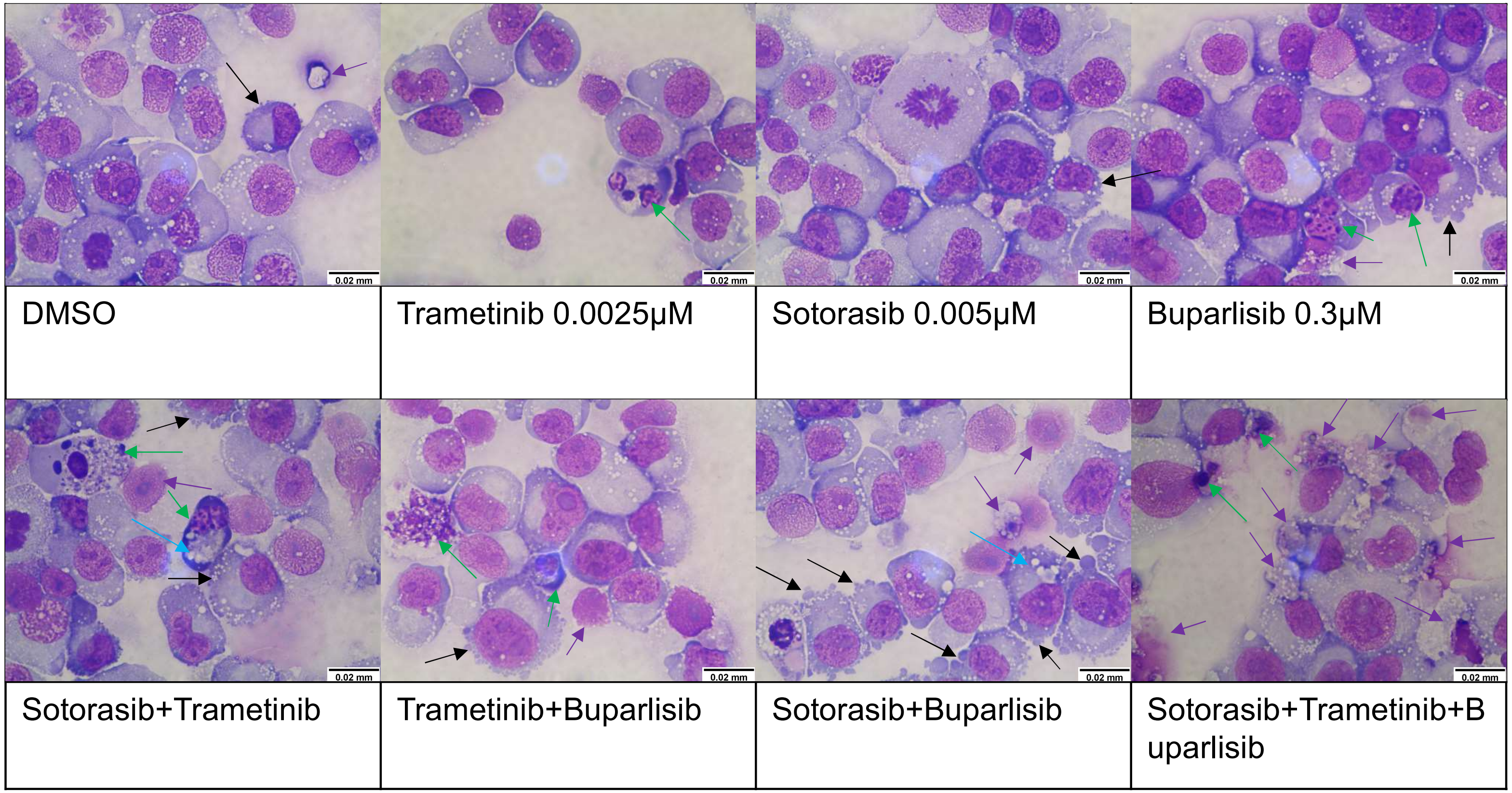

Supplementary Figure S5-5-3. Morphology changes of MIA PACA-2 after 72 hours Sotorasib, Trametinib, Buparlisib and inhibitor combination exposure (3rd). Magnification: 100×

↑ Membrane Bubbles, Membrane Bound Apoptotic Body, ↑ Vacuolization, ↑ Apoptotic Body, ↑ Nuclear Condensation/Fragmentation, ↑ Rupture of the Plasma Membrane

Supplementary Figure S5-5-4

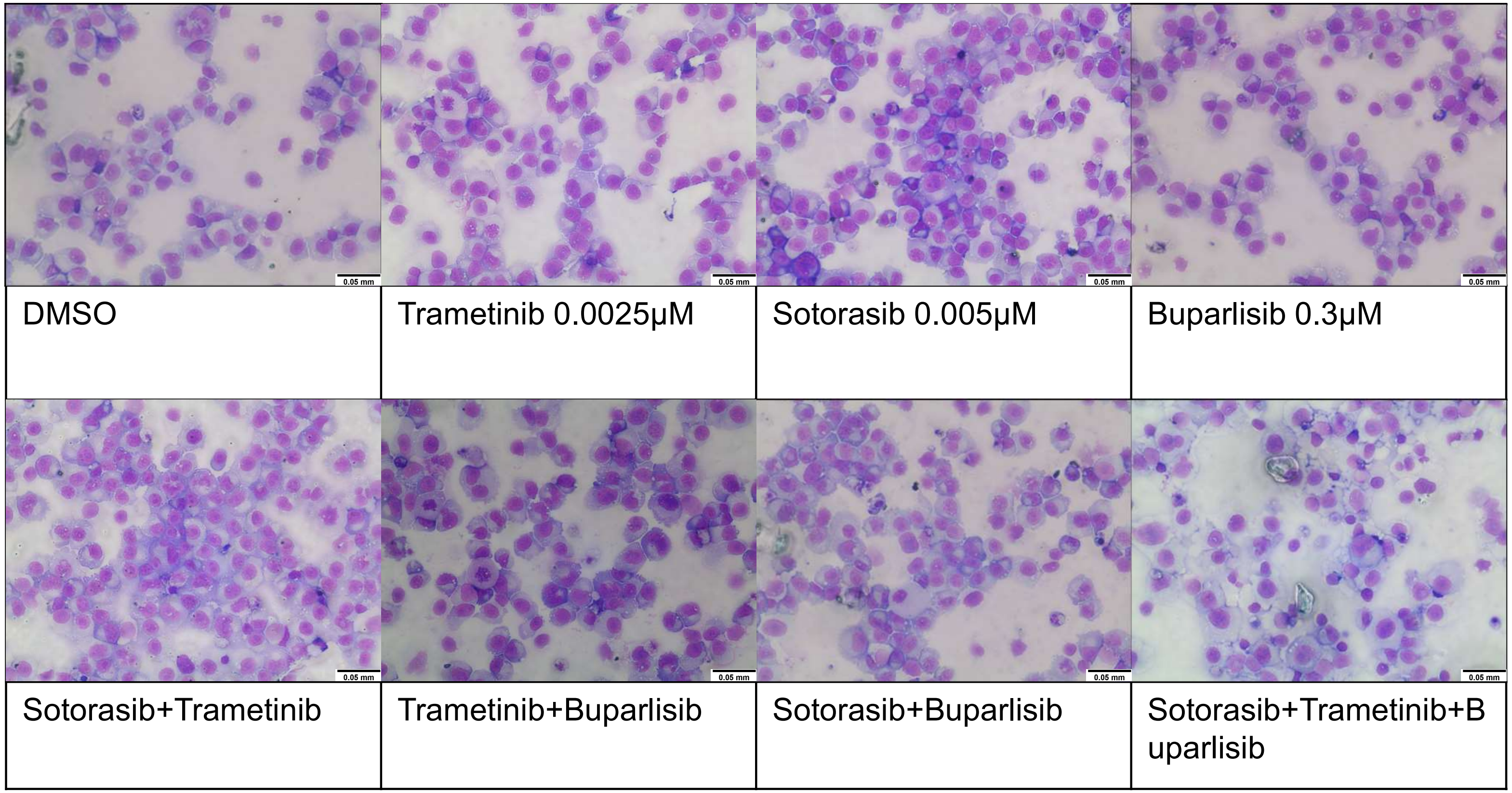

Supplementary Figure S5-5-4. Morphology changes of MIA PACA-2 after 72 hours Sotorasib, Trametinib, Buparlisib and inhibitor combination exposure (1st). Magnification: 40×

Supplementary Figure S5-5-5

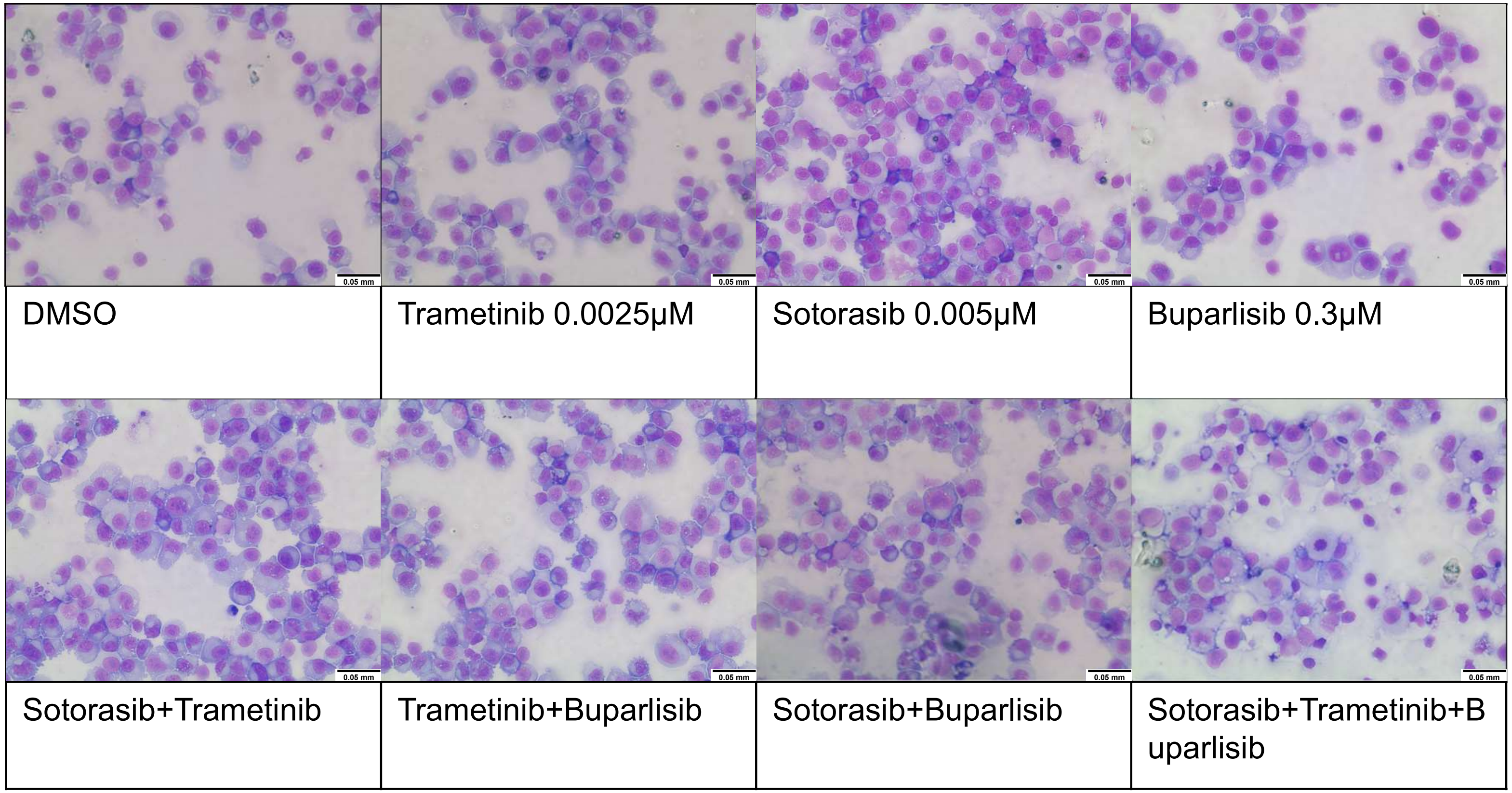

Supplementary Figure S5-5-5. Morphology changes of MIA PACA-2 after 72 hours Sotorasib, Trametinib, Buparlisib and inhibitor combination exposure (2nd). Magnification: 40×

Supplementary Figure S5-5-6

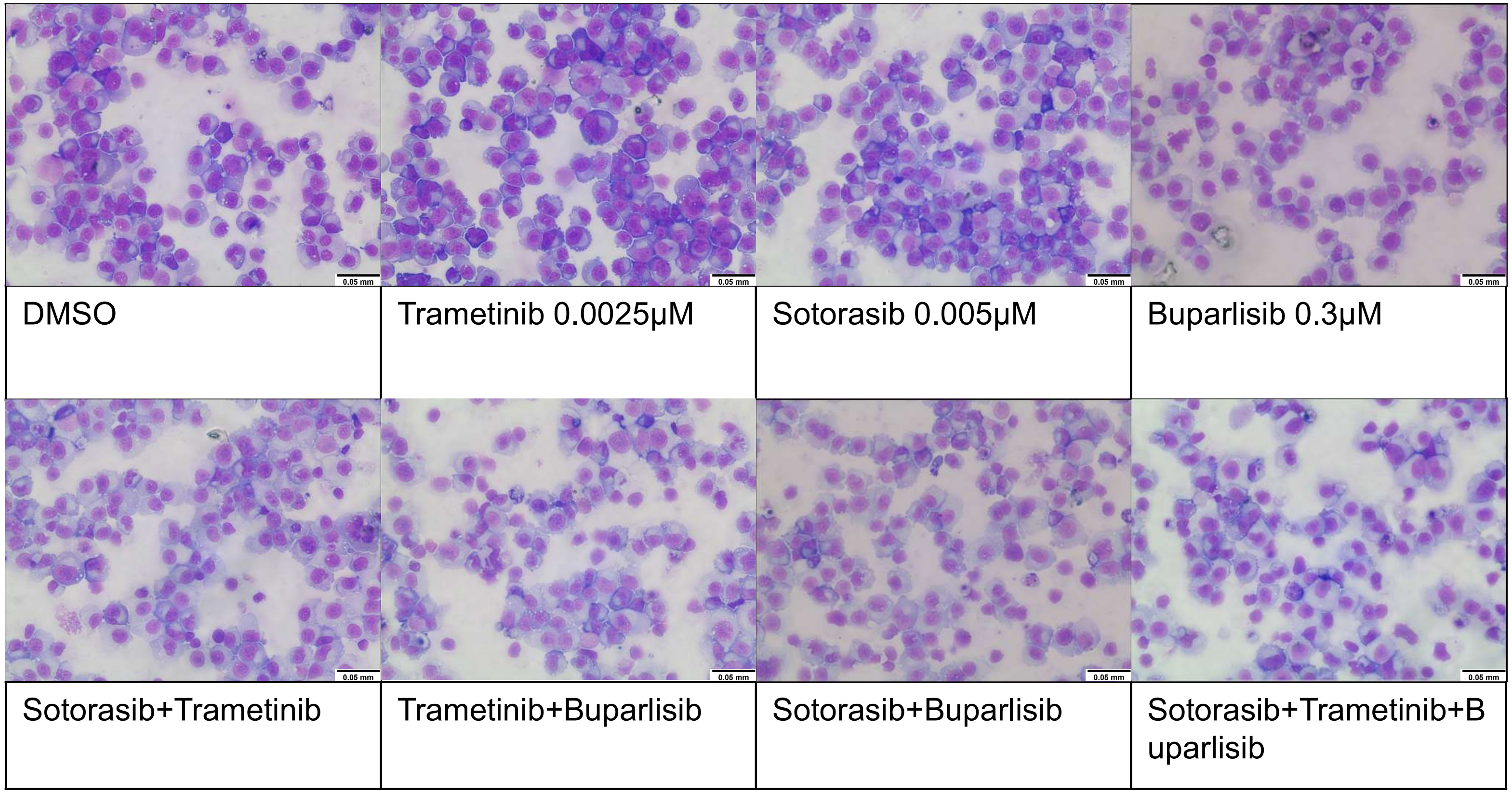

Supplementary Figure S5-5-6. Morphology changes of MIA PACA-2 after 72 hours Sotorasib, Trametinib, Buparlisib and inhibitor combination exposure (3rd). Magnification: 40×

Supplementary Figure S6

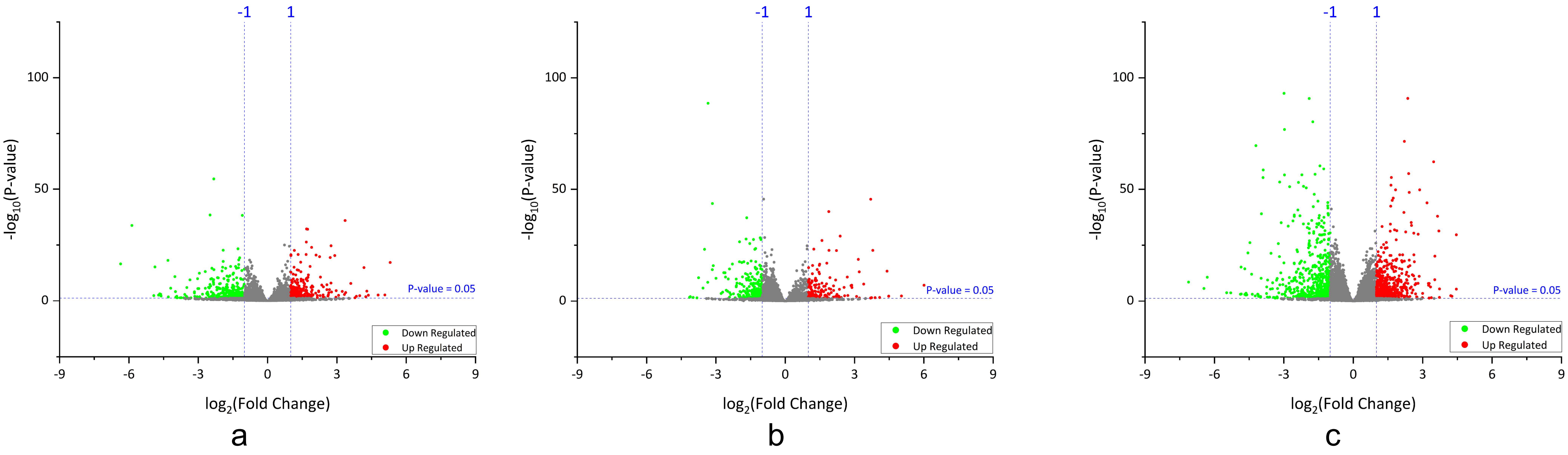

Supplementary Figure S6. Recognize of DEGs after ASPC-1 (a), BXPC-3 (b), CAPAN-1 (c) exposed to the combination of BI-3401, Trametinib and Buparlisib.

Supplementary Figure S7

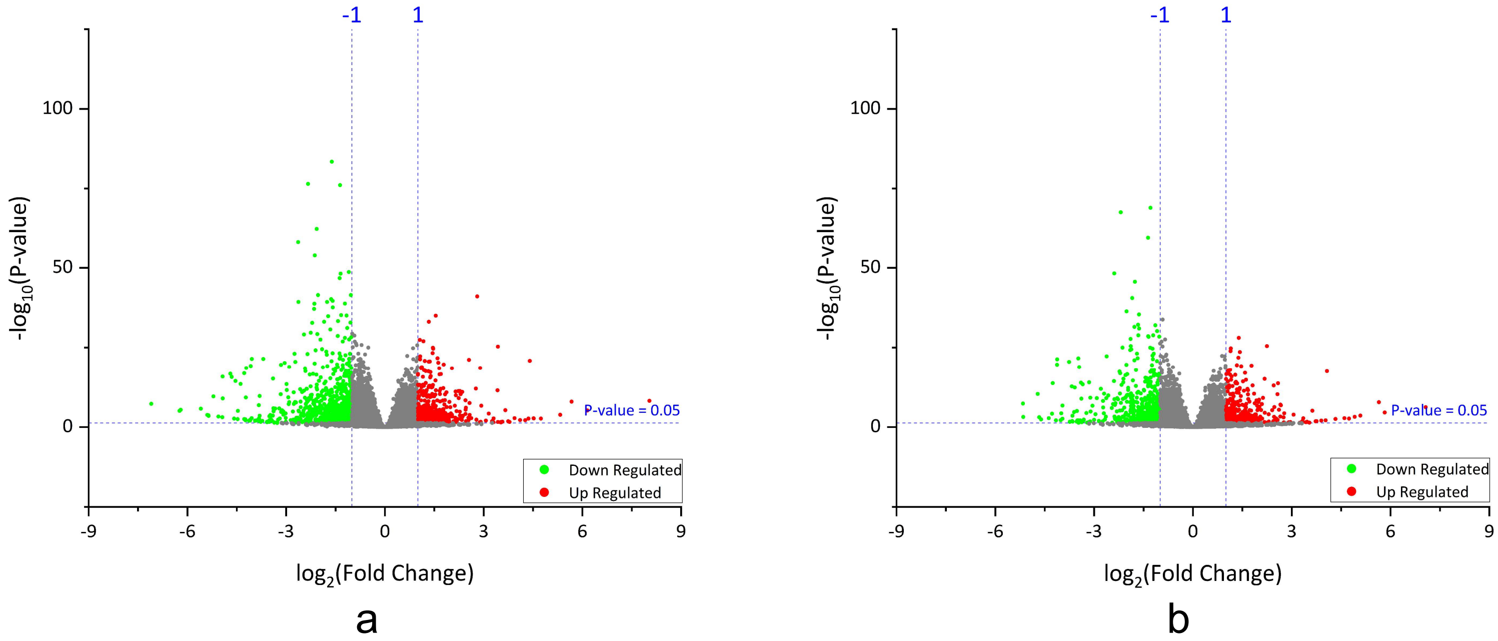

Supplementary Figure S7. Recognize of DEGs after MIA PACA-2 exposed to the combination of BI-3401, Trametinib and Buparlisib (a) or Sotorasib, Trametinib and Buparlisib (b).

Supplementary Figure S8

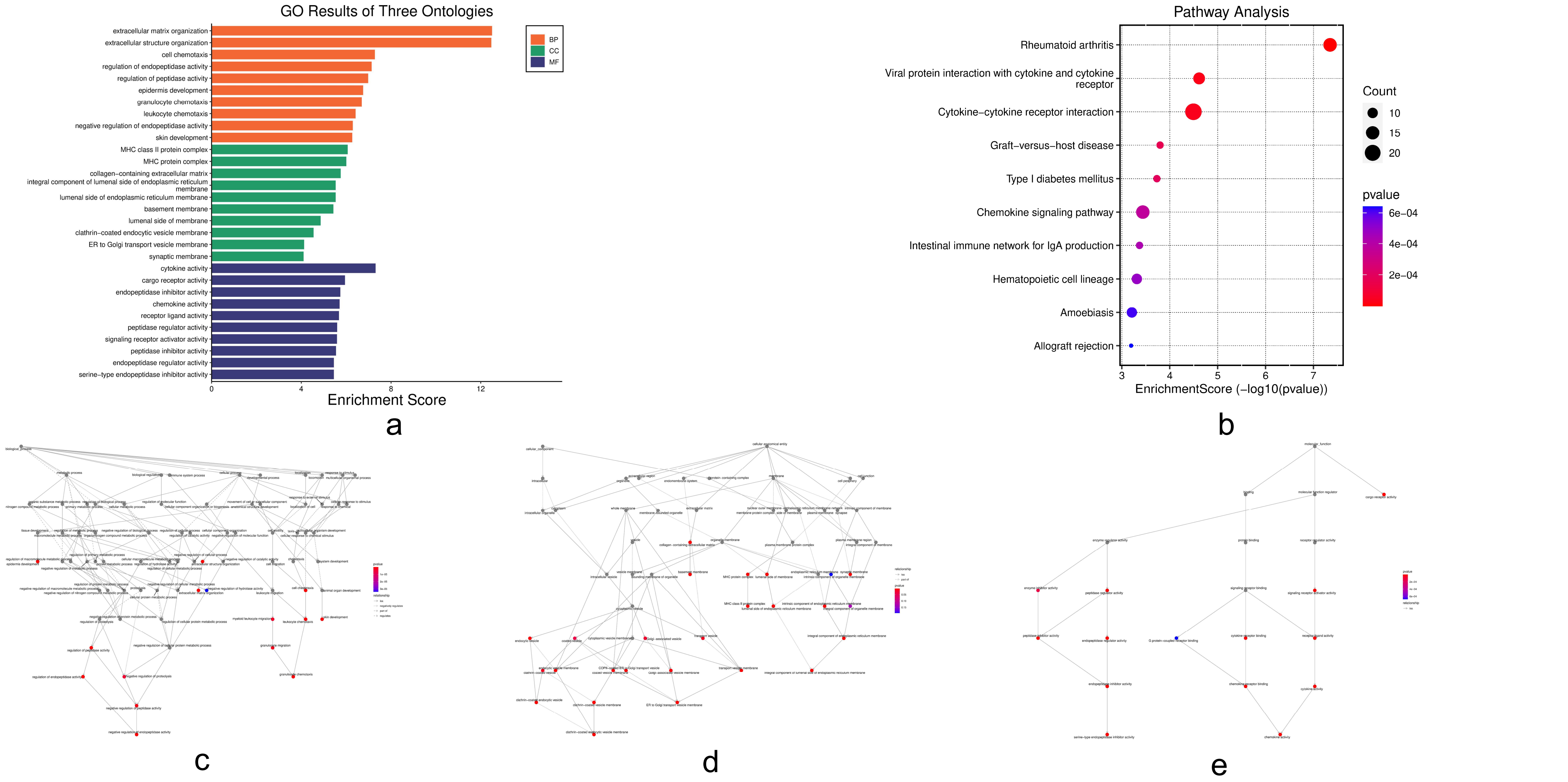

Supplementary Figure S8. The top 10 results of DEGs enrichment in three gene ontology (a) and KEGG pathway (b), as well as go plot BP (c), CC (d), and MF (e) in ASPC-1 after BI-3406+Trametinib+Buparlisib exposure. BP: biological process, CC: cellular functions, MF: molecular functions.

Supplementary Figure S9

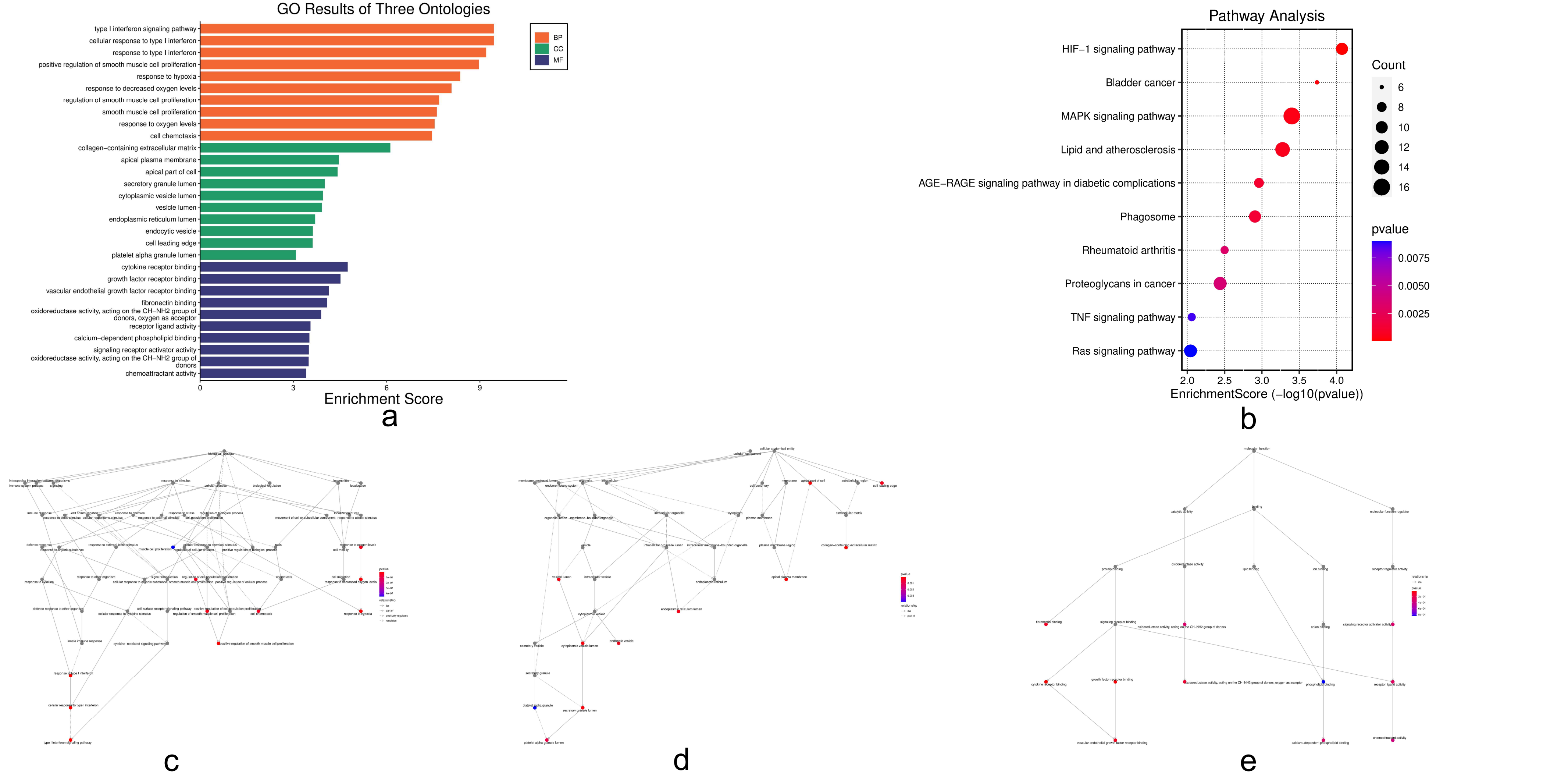

Supplementary Figure S9. The top 10 results of DEGs enrichment in three gene ontology (a) and KEGG pathway (b), as well as go plot BP (c), CC (d), and MF (e) in BXPC-3 after BI-3406+Trametinib+Buparlisib exposure. BP: biological process, CC: cellular functions, MF: molecular functions.

Supplementary Figure S10

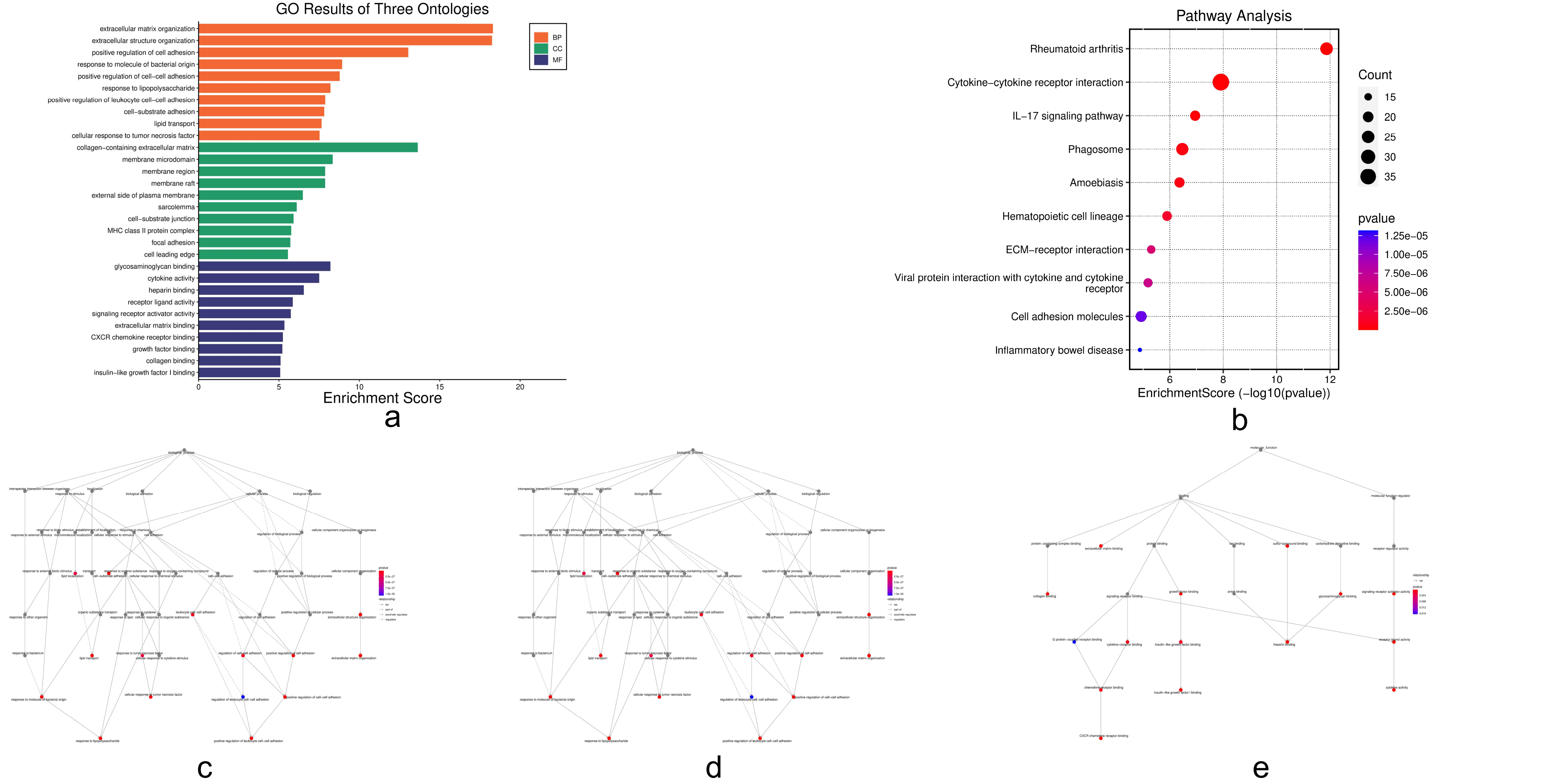

Supplementary Figure S10. The top 10 results of DEGs enrichment in three gene ontology (a) and KEGG pathway (b), as well as go plot BP (c), CC (d), and MF (e) in CAPAN-1 after BI-3406+Trametinib+Buparlisib exposure. BP: biological process, CC: cellular functions, MF: molecular functions.

Supplementary Figure S11

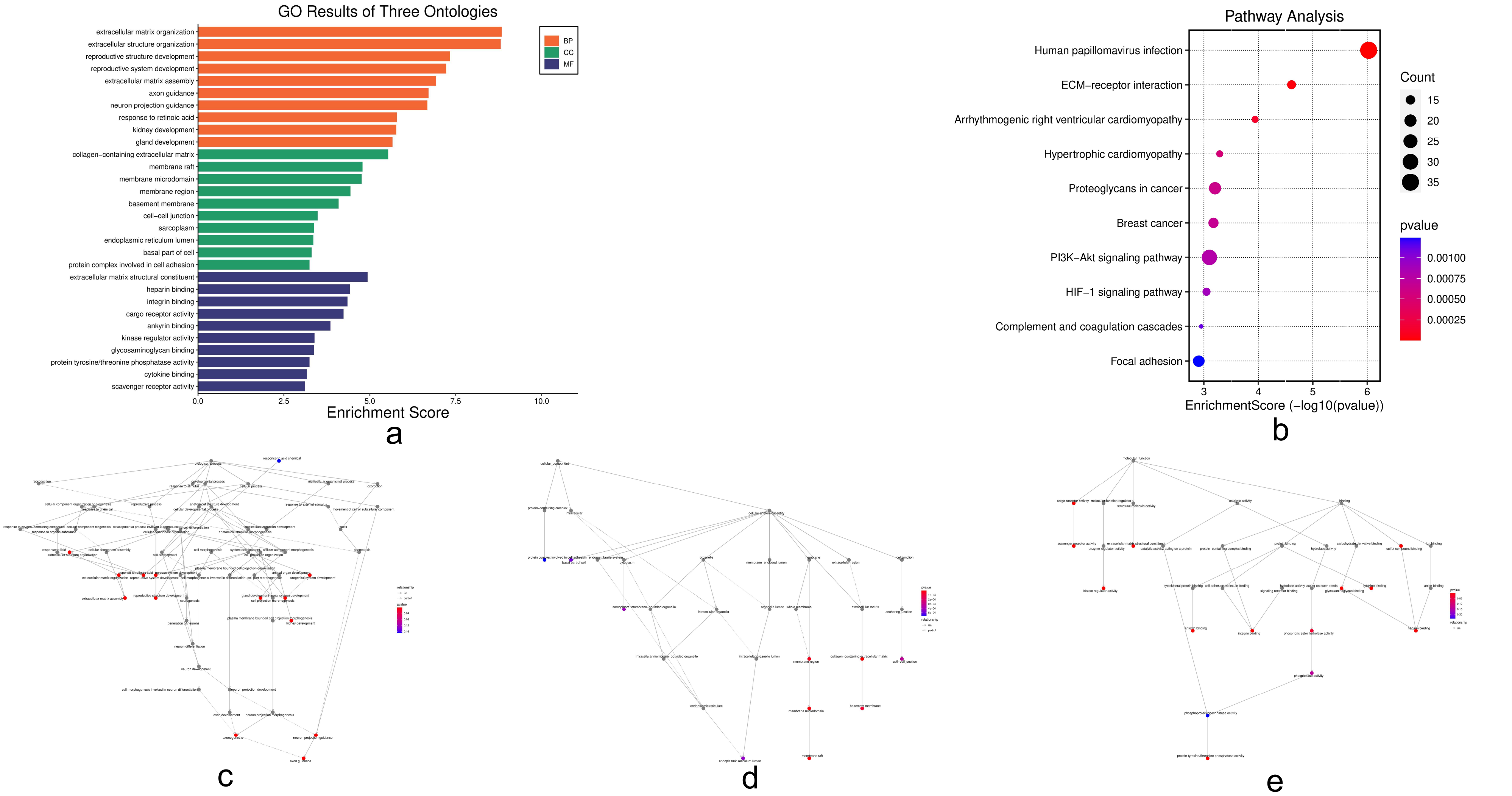

Supplementary Figure S11. The top 10 results of DEGs enrichment in three gene ontology (a) and KEGG pathway (b), as well as go plot BP (c), CC (d), and MF (e) in MIA PACA-2 after BI-3406+Trametinib+Buparlisib exposure. BP: biological process, CC: cellular functions, MF: molecular functions.

Supplementary Figure S12

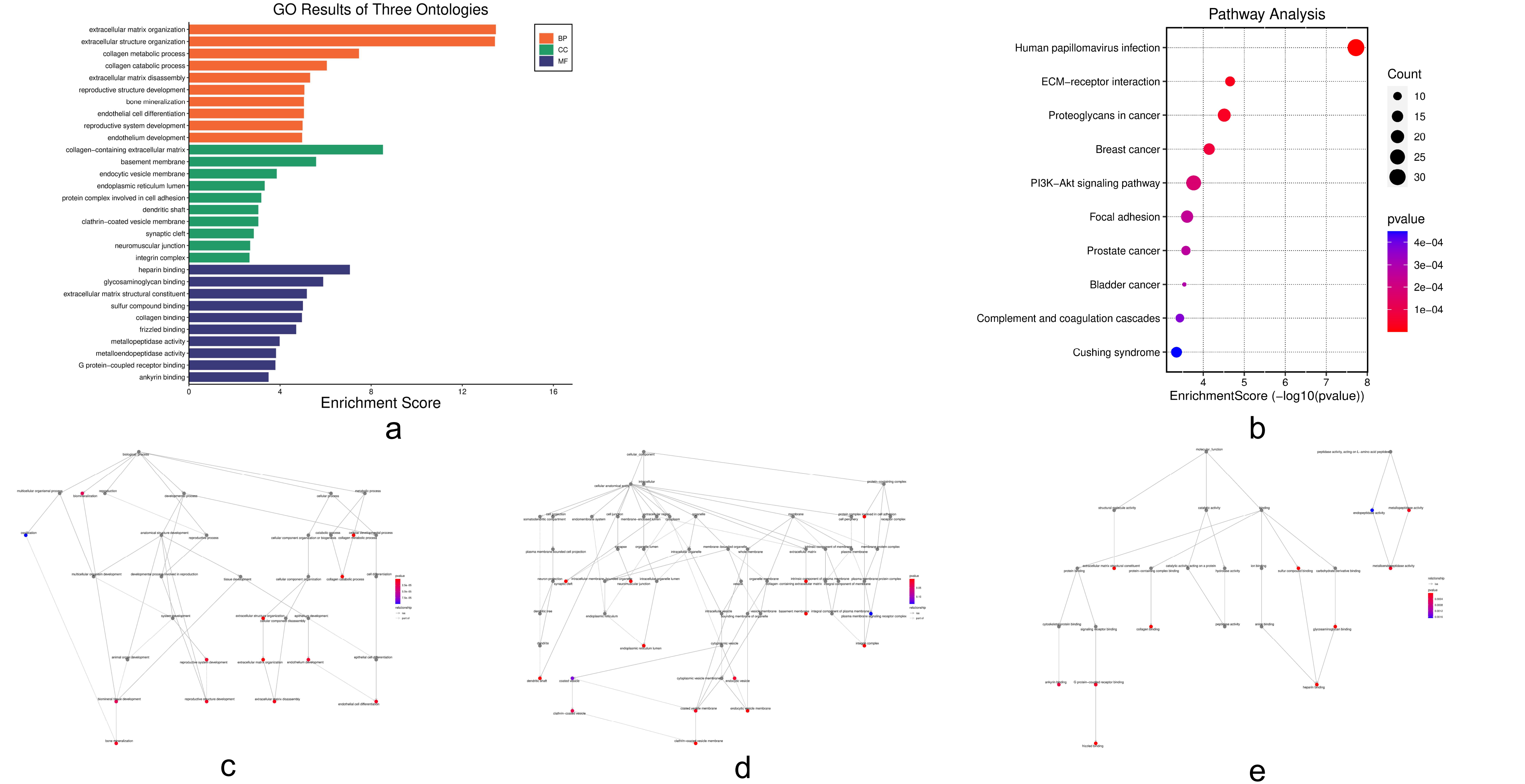

Supplementary Figure S12. The top 10 results of DEGs enrichment in three gene ontology (a) and KEGG pathway (b), as well as go plot BP (c), CC (d), and MF (e) in MIA PACA-2 after Sotorasib+Trametinib+Buparlisib exposure. BP: biological process, CC: cellular functions, MF: molecular functions.
